# The dynamics of biomarkers across the clinical spectrum of Alzheimer’s disease

**DOI:** 10.1186/s13195-020-00636-z

**Published:** 2020-06-13

**Authors:** Christoforos Hadjichrysanthou, Stephanie Evans, Sumali Bajaj, Loizos C. Siakallis, Kevin McRae-McKee, Frank de Wolf, Roy M. Anderson, the Alzheimer’s Disease Neuroimaging Initiative

**Affiliations:** 1 Department of Infectious Disease Epidemiology, School of Public Health, Imperial College London, London, UK; 2 Modelling and Economics Unit, National Infection Service, Public Health England, London, UK; 3 Lysholm Department of Neuroradiology, National Hospital for Neurology and Neurosurgery, University College London Hospitals, London, UK

**Keywords:** Alzheimer’s disease, Dementia, Biomarker trajectories, Clinical states, Time to event, Markov chain, ADNI, CSF, PET, Plasma, Brain

## Abstract

**Background:**

Quantifying changes in the levels of biological and cognitive markers prior to the clinical presentation of Alzheimer’s disease (AD) will provide a template for understanding the underlying aetiology of the clinical syndrome and, concomitantly, for improving early diagnosis, clinical trial recruitment and treatment assessment. This study aims to characterise continuous changes of such markers and determine their rate of change and temporal order throughout the AD continuum.

**Methods:**

The methodology is founded on the development of stochastic models to estimate the expected time to reach different clinical disease states, for different risk groups, and synchronise short-term individual biomarker data onto a disease progression timeline. Twenty-seven markers are considered, including a range of cognitive scores, cerebrospinal (CSF) and plasma fluid proteins, and brain structural and molecular imaging measures. Data from 2014 participants in the Alzheimer’s Disease Neuroimaging Initiative database is utilised.

**Results:**

The model suggests that detectable memory dysfunction could occur up to three decades prior to the onset of dementia due to AD (ADem). This is closely followed by changes in amyloid-β CSF levels and the first cognitive decline, as assessed by sensitive measures. Hippocampal atrophy could be observed as early as the initial amyloid-β accumulation. Brain hypometabolism starts later, about 14 years before onset, along with changes in the levels of total and phosphorylated tau proteins. Loss of functional abilities occurs rapidly around ADem onset. Neurofilament light is the only protein with notable early changes in plasma levels. The rate of change varies, with CSF, memory, amyloid PET and brain structural measures exhibiting the highest rate before the onset of ADem, followed by a decline. The probability of progressing to a more severe clinical state increases almost exponentially with age. In accordance with previous studies, the presence of apolipoprotein E4 alleles and amyloid-β accumulation can be associated with an increased risk of developing the disease, but their influence depends on age and clinical state.

**Conclusions:**

Despite the limited longitudinal data at the individual level and the high variability observed in such data, the study elucidates the link between the long asynchronous pathophysiological processes and the preclinical and clinical stages of AD.

## Background

Alzheimer’s disease (AD) is a neurodegenerative disorder with long preclinical and prodromal stages [1, 2], where pathophysiological changes, and potentially irreversible brain damage, occur many years prior to clinical manifestation. Despite the problems and practicalities of collecting frequent biomarker data over a long period of time, research over the last two decades has improved our understanding of the natural history of AD and demonstrated the importance of considering the disease as a multifaceted process moving along a biological and clinical continuum [3].

To date, there is limited longitudinal data for individuals with repeated biomarker measures throughout the AD continuum. Hence, descriptions of the continuous, temporal dynamics of potential biological and clinical markers during the progression of individuals from healthy stages to dementia due to AD (ADem) are mainly based on highly heterogeneous data collected over a relatively short period of time. Previous work has mainly focused on the description of the dynamics within different clinical states [4, 5]. In studies of autosomal dominant AD, early pathologic changes can be described as a function of the expected time to symptoms onset. This time is predicted based on the age of the individual and a retrospective estimate of the age at which the parent developed AD symptoms [6]. Estimates in these studies have also been produced based on the mean age at onset of all other individuals with the same autosomal dominant AD mutation type [7]. In the common form of sporadic AD, estimating the time required to reach a disease state is more challenging [8]. Techniques that have been employed for the prediction of the dynamics of biomarkers include the synchronisation of individual data based on a disease progression score, which can be a function of the individual’s age [9, 10], and the optimal alignment of individuals’ biomarker trajectories with the estimated long-term population-level trajectory [11, 12]. Time-dependent changes of potential biomarkers have also been characterised on different scales of disease progression based on the individual biomarker rate of change as estimated from the short-term longitudinal observations [2, 13–15]. Other data-driven progression models have recently emerged for the description of disease progression without the need of a priori staging of individuals [13].

Although the description of the evolution of potential biomarkers without relying on pre-defined clinical stages has its own merits, the diagnosis of AD based exclusively on the presence of abnormal levels of specific biomarkers is still under research [16]. On the other hand, categorising individuals using clinical syndromic criteria is useful in clinical practice [17]. Despite the challenges and limitations, this categorisation has been helpful in clinical and epidemiological studies, including the evaluation and standardisation of individuals’ clinical state and the assessment of the public health impact and cost of care.

In this study, we aim to describe the pathophysiological changes that may be observed during the clinical progression from cognitively healthy stages to Alzheimer’s clinical syndrome [16] and provide further insights into their temporal relation. Based on the clinical diagnosis and a set of demographic and genetic factors, we develop a stochastic model to estimate the expected time required for an individual at a specific clinical state to reach any of the more severe states. Three commonly accepted clinical states are considered: the cognitively normal state (CN), the mild cognitive impairment state (MCI) and the ADem state [16, 18]. Two established major risk factors for sporadic Alzheimer’s are taken into account: the presence of the apolipoprotein E *ɛ*4 genotype (APOE *ɛ*4) [19] and age. We also extend the model to consider the effect of educational attainment [20]. Individual data from a range of cognitive and pathologically relevant disease markers are aligned based on their temporal distance from a disease state. Assuming that similar biological and cognitive changes occur in all individuals within a defined risk group, the population-level dynamics of markers and a temporal order of changes are estimated. The focus is on the link between dynamic pathophysiological changes and the clinical presentation of various clinical stages of AD.

## Methods

### Clinical progression

#### The model

We developed a Markov model to estimate the expected time needed for different risk groups to reach a given clinical state, provided individuals will eventually develop ADem, i.e. that death is not a competing event and ADem is the only absorbing state.

**Fig. 1:**
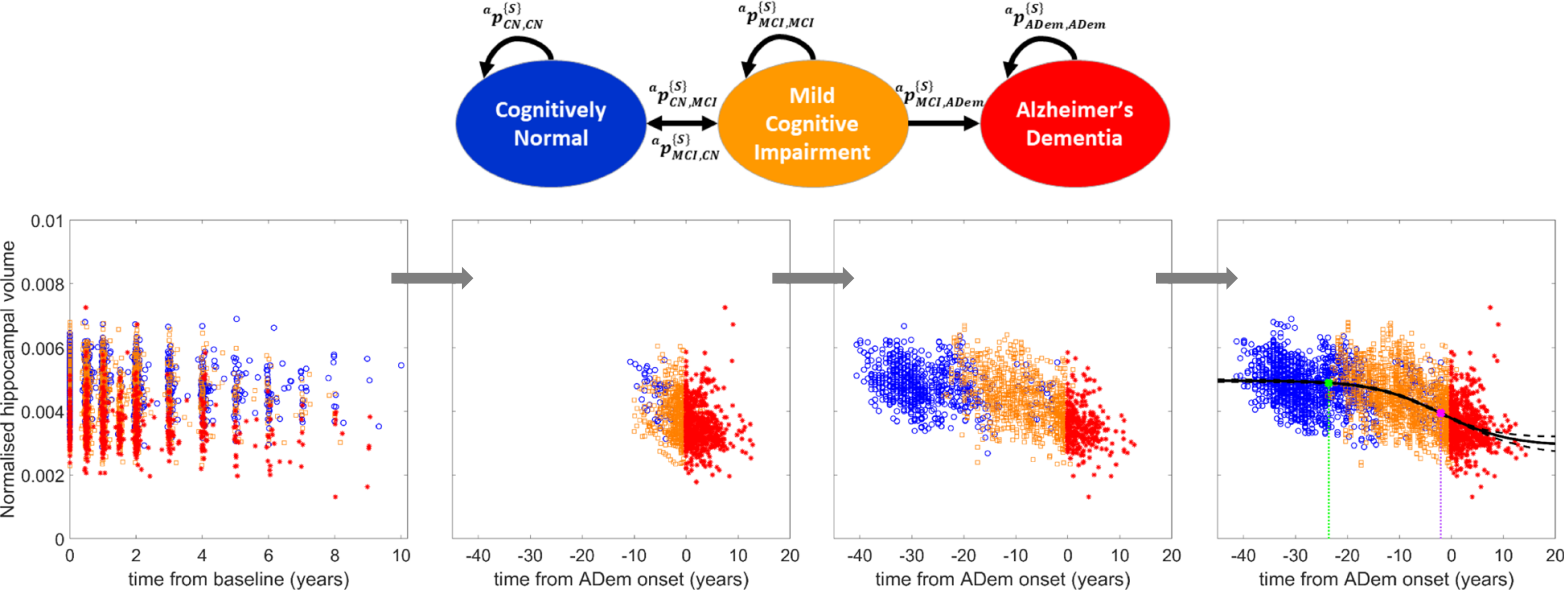
Example illustrating the procedure for the development of biomarker trajectories. In the first step, the observed data is collected and the characteristics of individuals are considered. In the second step, those individuals that have transitioned to ADem during the study are aligned on a disease progression timeline, where time 0 is the time of the first ADem diagnosis. In the third step, for every individual that has not developed ADem symptoms during the study, the Markov model is used to estimate the expected time (number of years) until ADem; given their demographic and genetic characteristics (here, their age and APOE ε4 status), the expected time to ADem diagnosis is estimated using the formulae derived analytically (Additional file 1: Section S2). The distributions of the 1-year transition probabilities used in the Markov model have been estimated from a generalised linear mixed model (see the ‘Transition probabilities’ section and Additional file 1: Section S1) using the Gibbs sampler (the ‘Statistical analysis’ section). In the last step, a sigmoid function (linear for Aβ_1–40_, Aβ_1–42_ and t-tau markers in plasma) is fitted to each biomarker data using non-linear least square estimation. The time point at which the first significant biomarker change occurs (green) is defined as the first point at which the 95% CI of the mean biomarker level does not overlap with the 95% CI of the initial mean biomarker level. The 95% CI of the best fit was estimated using the delta method. The inflection point (purple) is the point at which the maximum biomarker rate of change is reached, that is, the point at which the second derivative of the best fit is equal to 0

Let ^*a*^*I*^{*S*}^ denote the group of individuals at age *a* that have a set of time-invariant characteristics {*S*}. Individuals ^*a*^*I*^{*S*}^ that have been clinically classified in state A can transition to state B within the next year with probability ${}^a{p}_{A,B}^{\left\{S\right\}}$. The possible transitions between the three clinical states, CN, MCI and ADem, are as shown in the schematic diagram in Fig. 1 [21, 22].

### Transition probabilities

Generalised linear mixed models (GLMMs) with a logit link function were developed and fitted, and then the probability of transitioning from state A to state B was estimated conditional on the covariates [23]. The main model incorporated four covariates, namely, age (age-squared) at the point of transition to a clinical state (continuous, centred at 50 years and scaled by a factor of 100 to normalise), APOE ε4 status (binary, presence/absence of APOE ε4 alleles), time between clinic visits in years (centred at 12 months) and the current diagnostic state (CN, MCI, ADem). An interaction term between age-squared and diagnostic state was also included. We also explored the significance of an interaction term between APOE ε4 status and age in the model. While several studies have reported that APOE ε4-related risk for developing ADem may vary significantly with age [24, 25], in the sample dataset we considered it has been shown that this term is not significant (*p* > 0.7, checked at the 5% level of significance) and the model without the interaction term provides better fits to the data. When the educational level was incorporated into the model, this was defined dichotomously based on the number of years of education; ≤16 or >16. The choice of 16 years as the cut-off point in education has been made based on the median (= 16) and mean (= 16.04) of the years of education in the sample. Details of the GLMM are provided in Additional file 1: Section S1.

### Expected time to MCI and ADem

Given the demographic and genetic characteristics of individuals at the time point of each measurement, and the respective transition probabilities from that point, the expected time required to reach a more severe state was estimated using the formulae derived from the stochastic model, presented in Additional file 1: Section S2.

### Marker trajectories

#### Development

We assumed that during the development and progression of the disease, all individuals in a defined risk group (see the ‘Dataset: the Alzheimer’s Disease Neuroimaging Initiative’ section) present similar cognitive and biological dynamic changes. First, the measurements from individuals that have developed ADem during the study were aligned on a disease progression timeline, where time 0 is the time of ADem onset (visit at which ADem was first diagnosed). In order to provide insight into the behaviour of the markers after ADem onset, measurements of individuals that were diagnosed with ADem at their first visit have also been included by using the best estimate of the year of onset of ADem symptoms as reported in the data (years from ADem onset ≈ exam date – estimated date of onset, available in ADNI-1 and ADNI-GO). Following the calculation of the expected time to ADem from each measurement of CN and MCI individuals that have not developed ADem during the study, all their biomarker data were also aligned on the same disease timeline (see Fig. 1). Based on conceptual models and current evidence in the literature [9, 26], it was assumed that markers are monotonically decreasing or increasing during the disease progression following a non-linear temporal course, and in particular, a sigmoidal type pattern. This excludes the Aβ_1–40_, Aβ_1–42_ and total tau (t-tau) markers in plasma which, due to small changes during the clinical progression, were described by a linear model of the form *B*(*t*_ADem_) = *r*_1_ + *r*_2_*t*_ADem_, where *t*_ADem_ (independent variable) is the time from ADem, and *t*_ADem_ = 0 corresponds to the time of the first ADem diagnosis. A sigmoid function of the following form was fitted to each dataset for each other marker *B*:

1 $$ B\left({t}_{\mathrm{ADem}}\right)={r}_1+\frac{r_2-{r}_1}{1+\exp \left({r}_3\left({t}_{\mathrm{ADem}}-{r}_4\right)\right)}. $$

We restricted *r*_3_ < 0, so *r*_1_ is the value that *B*(*t*_ADem_) approaches to as *t*_ADem_ →  − ∞, and *r*_2_ is the value that *B*(*t*_ADem_) approaches to as *t*_ADem_ → ∞.

### Normalisation

Changes in the potential AD markers have been considered collectively on the same scale by using the min-max normalisation method, so that the normalised form, *B*_norm_, of the mean trajectory *B* is given by:

2 $$ {B}_{\mathrm{norm}}\left({t}_{\mathrm{ADem}}\right)=\left|\frac{B\left({t}_{\mathrm{ADem}}\right)-\underset{t_{\mathrm{ADem}}\to -\infty }{\lim }B\left({t}_{\mathrm{ADem}}\right)}{\underset{t_{\mathrm{ADem}}\to -\infty }{\lim }B\left({t}_{\mathrm{ADem}}\right)-\underset{t_{\mathrm{ADem}}\to +\infty }{\lim }B\left({t}_{\mathrm{ADem}}\right)}\right|. $$

$\underset{t_{\mathrm{ADem}}\to -\infty }{\lim }{B}_{\mathrm{norm}}\left({t}_{\mathrm{ADem}}\right)=0$ is thus the healthiest level (normal), and $\underset{t_{\mathrm{ADem}}\to +\infty }{\lim }{B}_{\mathrm{norm}}\left({t}_{\mathrm{ADem}}\right)=1$ is the most severe level (abnormal). The limits of *B*(*t*_ADem_), $\underset{t_{\mathrm{ADem}}\to -\infty }{\lim }B\left({t}_{\mathrm{ADem}}\right)$ and $\underset{t_{\mathrm{ADem}}\to +\infty }{\lim }B\left({t}_{\mathrm{ADem}}\right)$ are fixed to the respective limits of the best fit of *B*(*t*_ADem_).

### First biomarker change, rate of change and inflection point

We considered the time of the first significant biomarker change to be the first time point at which the 95% confidence interval (CI) of the predicted mean biomarker level does not overlap with the 95% CI of the predicted mean value before any change (the lowest or the highest mean value, depending on whether the biomarker monotonically increases or decreases). For each normalised marker trajectory, the instantaneous rate of change, which represents the change in the marker level at each instant in time, and the inflection point, which is the point at which the maximum rate is observed before start declining, have also been considered. These were derived by calculating the first and the second derivative, respectively, of the equation describing the trajectory.

### Markers considered

We focused our study on a number of markers that, alone or with other markers, could serve as indicators of AD progression and/or endpoints in clinical trials. Twenty-seven markers have been considered in total. The markers can be classified into four main categories: markers of cognitive performance, biological markers that can be measured in cerebrospinal (CSF) and plasma fluid, and neuroimaging markers.

#### Cognitive markers

Two AD Assessment Scale-Cognitive Subscale tests which serve as AD and dementia indicators: the ADAS-Cog 11 (ranged from 0 to 70, with the first number being the least severe and the last the most severe [27]) and the ADAS-Cog 13 (0 to 85) [28]; the Mini-Mental State Examination (MMSE) test (30 to 0) [29] which is an indicator of mental status; the Clinical Dementia Rating Scale-Sum of Boxes (CDR-SB) (0 to 18) [30, 31]; the Montreal Cognitive Assessment (MoCA) (30 to 0) [32]; and the Functional Activities Questionnaire (FAQ), concerned with performing daily tasks necessary for independent living (0 to 30, with higher scores reflecting greater dependency) [33]. Two efficient neuropsychological instruments for evaluating different aspects of episodic memory have also been considered: the Rey’s Auditory Verbal Learning Test (RAVLT) [34] (here, we focused on RAVLT-Immediate recall scores (RAVLT_Immediate, 75 to 0)), and the Logical Memory-Delayed recall scores (LM_Delayed, 25 to 0) [35]. Additionally, we investigated the change in two composite scores, each of which is a weighted combination of components of well-validated neuropsychological tests. The first is the Preclinical Alzheimer Cognitive Composite [36] (PACC, the modified version of [37] to include the Trail Making Test B), which has been designed to serve as the primary outcome measure for trials conducted at the asymptomatic phase of the disease (lower scores indicate more severe impairment). The second is the AD Composite Score (ADCOMS) (0 to 1.97) [38], which has been developed to detect a related clinical decline in amnestic MCI due to AD, and prodromal and mild AD.

#### CSF markers

The 42-amino acid form of amyloid β protein, Aβ_1–42_, which indicates brain amyloid pathology [39]; the neurofilament light (NfL), a biomarker of neuroaxonal injury or neurodegeneration [40]; the total tau (t-tau) and phosphorylated tau (p-tau) proteins [39], which can be considered as potential biomarkers of Alzheimer-type axonal degeneration and neurofibrillary tangle formation, respectively; and neurogranin (Ng), which has also recently emerged as a potential synaptic biomarker [41].

#### Plasma fluid markers

Aβ_1–40_; Aβ_1–42_; NfL; t-tau.

#### Neuroimaging markers

The MRI volumetric measurements of the following brain regions have been considered: hippocampus, whole brain, entorhinal cortex, fusiform gyrus and middle temporal gyrus [42]. To account for the inter-individual variation in head size, all volumes of interest have been normalised for total intracranial volume (ICV) [43], producing a unitless value between 0 and 1. We also considered ^18^F-fluorodeoxyglucose (FDG) positron emission tomography (PET) which is used to visualise changes in the cerebral glucose metabolism [44], and standardised uptake value ratio (SUVr) for Pittsburgh compound B (PiB)-PET [45] and florbetapir (F18-AV-45) PET [46], which both allow in vivo assessment of cerebral amyloid load.

### Dataset: the Alzheimer’s Disease Neuroimaging Initiative

The dataset used was obtained from the Alzheimer’s Disease Neuroimaging Initiative (ADNI) database (adni.loni.usc.edu, last downloaded on September 1, 2018). ADNI is a multisite (63 ADNI clinical trial sites are located across the USA and Canada) longitudinal study of normal cognitive ageing, MCI and early AD individuals. It has four phases: ADNI-1, ADNI-GO, ADNI-2 and ADNI-3. ADNI is not a population-based study; its population represents a primarily amnestic clinical population that can be used in treatment trials. Participants were recruited following specific recruitment methods. The procedure of ADem clinical diagnosis follows the standardised clinical criteria as outlined by the NINCDS-ADRDA guidelines [18]. For a detailed list of all diagnostic, and inclusion and exclusion criteria, readers are referred to the Procedures Manual (http://adni.loni.usc.edu/methods/documents).

The original dataset obtained from ADNI consists of 2066 individuals. The study duration up to the time the current analysis was performed is about 12 years. Repeated assessments took place every 6 months for the first 2 years and yearly afterwards. Two hundred fifty-five participants (from ADNI-2) were categorised as ‘significant memory concern’ (SMC) at baseline, and in the current analysis, were combined with individuals diagnosed as CN; the key inclusion criteria that distinguish SMC are a self-report significant memory concern from the participant, although participants in this class scored within the normal range for cognition (or CDR  =  0). However, this is not equated as progressive memory impairment or as consistent forgetfulness. In addition, in ADNI-2, MCI is divided further into ‘early MCI’ (EMCI) and ‘late MCI’ (LMCI), and in this analysis, the MCI subtypes were combined. Thirty-two individuals that had only a screening visit/diagnosis (preceding the baseline visit/diagnosis) were excluded from the analysis. Out of the 698 individuals that developed dementia, 20 developed non-Alzheimer’s dementias and were also removed.

For the prediction of clinical progression and calculation of the transition probabilities, we used only those individuals that had at least two visits with clinical diagnosis. For the development of a marker trajectory in a specific risk group (e.g. those that reached abnormal amyloid levels), all available individual-level measurements in that group were utilised, given that all information for the calculation of the expected time to ADem from the point of measurement was available. The trajectory of each marker was first estimated independently for the entire sample (Additional file 1: Section S3, Fig. S1); the ADNI dataset consists of a highly selective clinical population that can be considered as a good representation of a population that will most likely be affected by AD. The same method was then applied for only those individuals that have shown some evidence of substantial Aβ in their brain (amyloid-positive individuals), and so have an increased chance of being in the Alzheimer’s continuum [12, 16]. In particular, individuals that have ever crossed one of the following thresholds have been considered as amyloid-positive individuals: CSF Aβ_1–42_ = 880 pg/mL [47] or/and global PET florbetapir SUVr = 1.11, normalised to the whole cerebellum [48], or/and PiB-PET SUVr (standardised in the region relative to cerebellar grey matter) = 1.5 [2, 49, 50]. For the development of the trajectories in the subset of amyloid-positive individuals, the transition probabilities were re-calculated in this group.

To make a comparison of the times at which the first significant changes and the inflection points of biomarkers occur, the analysis was performed on a reduced dataset. The complete data for all biomarkers was not sufficient to support the model and provide good estimations for all biomarkers. As a compromise, we have chosen only the measurements at the visits where CSF Aβ_1–42_ was available; CSF Aβ_1–42_ is one of the markers with the lowest number of measurements, and many of the other markers have also been measured when CSF Aβ_1–42_ was measured (see Additional file 1: Section S3, Table S1). Data of CSF NfL and Ng, the plasma markers and PiB-PET could not always support the sigmoid function and have been excluded from the comparison with other markers of disease progression.

### Statistical analysis

The transition probabilities of the model were estimated using the Gibbs sampler, which is a Markov chain Monte Carlo (MCMC) algorithm. It was assumed that all parameters had improper uniform prior distributions, and convergence of the MCMC algorithms was assessed visually from the traces of each parameter. A number of GLMMs consisting of different combinations of variables have been considered. The model selection procedure was based on the deviance information criterion (DIC). The MCMC algorithms and goodness of fit statistics were implemented within the runMLwiN function from the R2MLwiN package in R [51].

The models of biomarker dynamics were fitted using iterative non-linear least square estimation, by initiating each step using the best parameter estimates of the previous step until the parameter estimates are optimised. When fitting the model, we took into account any known lower and upper limits of the markers, and we restricted *r*_1_, *r*_2_ and the prediction intervals accordingly so that the estimated values fall within these limits. For markers for which these limits are unknown, the lowest and highest values observed in the original dataset were used. The biomarker trajectories and all figures in the study were produced using MATLAB R2019a.

The 95% CI of all quantities have been computed using the 2.5th and 97.5th percentiles of the distribution obtained by performing the analysis on 500 bootstrap samples (produced by random sampling with replacement from the observations in the original sample used in each case, so that the number of measurements at each clinical state was equal to that of the original sample). For each bootstrap sample, probabilities of transitioning to different states were drawn at random from the respective probability distributions produced using the MCMC algorithm. The expected times to ADem onset were calculated for every individual in the sample, and the model of biomarker dynamics was fitted to it. The delta method was used to estimate the 95% CI of the best fit in each iteration of bootstrap resampling, which in turn was used for the calculation of the first significant biomarker change. For each marker, and each time point, we also estimated the 95% prediction interval for new observations (non-simultaneous bounds), which indicates the confidence that the new observation will lie within that interval given a single observation time point (predictor value). In addition to quantifying model uncertainty, further analysis has been performed to evaluate the performance of the model, including a single train-and-test experiment (holdout method), where the dataset of individuals that were CN at baseline and developed MCI and ADem during the study serves as the test set (Additional file 1: Section S7).

## Results

Individuals were removed from the dataset subject to the constraints described in the ‘Methods’ section, which yielded data from 2014 individuals. The baseline composition and characteristics of the population included in the analysis are presented in Table 1. Additional file 1: Section S3, Fig. S2 shows the age distribution of carriers and non-carriers of APOE ε4. During follow-up of these individuals, 7460 subsequent visits with clinical diagnosis following a previous diagnosis have been recorded (Table 2). Here, we present and focus on the results derived when utilising the entire sample independently of amyloid status. The change in the transition probabilities and expected time to ADem in amyloid-positive individuals, and the estimated biomarker trajectories in this group, are discussed in Additional file 1: Section S8. In addition, although we considered the influence of education and we present the results of its potential impact on the transition probabilities and expected time to ADem (see Additional file 1: Section S4, Fig. S3), due to the minor and, as yet, unclear effect on the biomarker dynamics, all the biomarker trajectories presented in this study have been developed based on the model that does not incorporate education.

**Table 1:** Dataset used. Number and characteristics of the 2014 individuals included in this analysis, by clinical diagnosis at baseline

		Clinical state at baseline		
		CN	MCI	ADem
Number of individuals				
Total		749	910	355
Chronological age at baseline (years)	≤ **70**	241	308	85
	**70 < age < 80**	402	423	170
	**> 80**	106	179	100
Gender	**Women**	413	370	157
	**Men**	336	540	198
Years of education	≤ **16**	385	537	247
	**> 16**	364	373	108
Genetic background (APOE ε4)	**Non-carriers of ε4 allele**	370	420	114
	**Carriers of 1 ε4 allele**	135	337	159
	**Carriers of 2 ε4 alleles**	12	92	65
Other characteristics (standard deviation of the mean in brackets)				
Average age at baseline (years)		73.20 (6.17)	73.04 (7.62)	75.03 (7.88)
Average number of years of education		16.55 (2.57)	15.93 (2.82)	15.24 (2.99)
Average time between visits (years)		1.02 (0.58)	0.8 (0.38)	0.64 (0.27)
Average follow-up (years)		3.36 (3.43)	3.69 (2.62)	1.38 (0.85)

**Table 2:** Number of transitions from one clinical state to another during follow-up

		Subsequent clinical diagnosis		
		CN	MCI	ADem
Clinical diagnosis	CN	2310	138	4
	MCI	104	3112	351
	ADem	0	26	1415

**Fig. 2:**
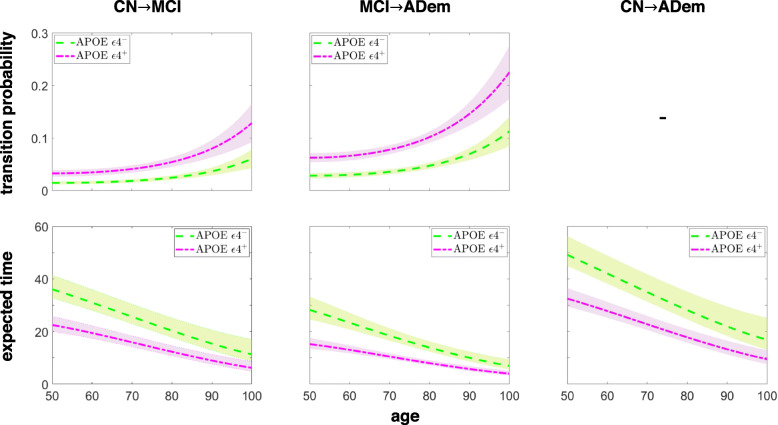
Probabilities and times to a more severe clinical state. Probabilities of transitioning to a more severe state within 1 year (top), and the expected time to reach a more severe state (bottom), as a function of age in the groups of carriers and non-carriers of APOE ε4. The shaded area represents the values within one standard deviation from the mean transition probabilities

The transition probabilities and the expected times to MCI and ADem onset as a function of age are illustrated in Fig. 2 for carriers and non-carriers of APOE ε4. It is observed that the probabilities increase almost exponentially with age, yielding a decrease in the required time to reach more severe clinical states. Depending on age and disease stage, the presence of APOE ε4 increases the likelihood and rate of progression. The time to ADem from the CN and MCI states is decreased by more than 10 and 5 years, respectively, when individuals aged 80 years or less are carriers of the APOE ε4 genotype.

### Biomarker trajectories

**Fig. 3:**
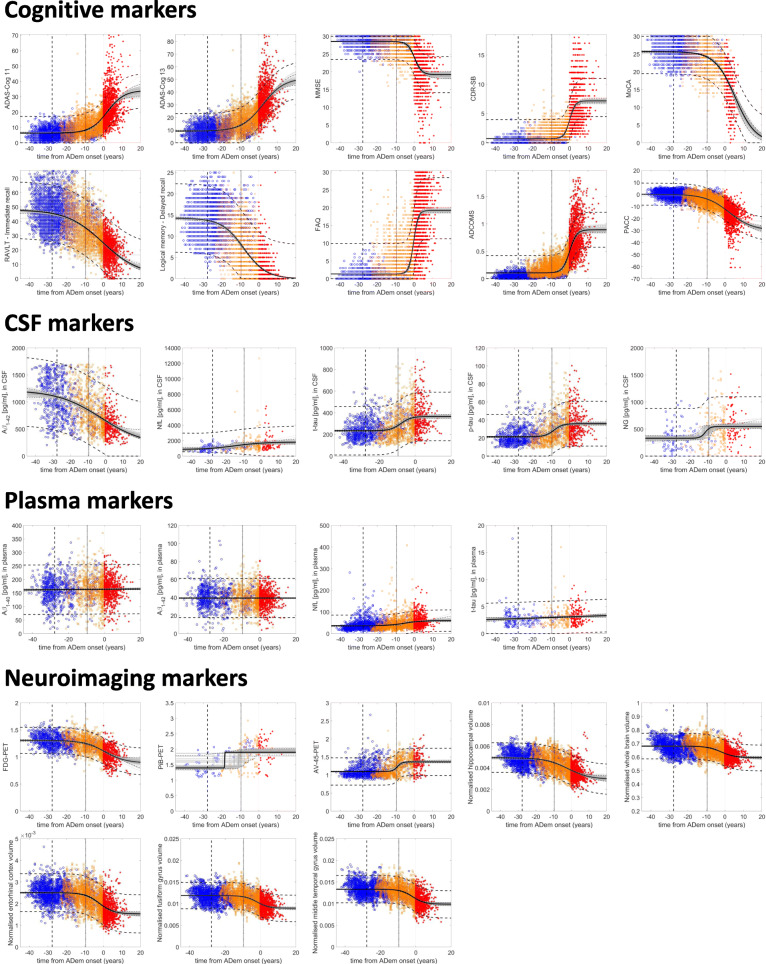
Trajectories of cognitive, CSF, plasma and neuroimaging markers. The black solid line represents the best fit of the sigmoid function (linear for Aβ_1–40_, Aβ_1–42_ and t-tau in plasma), the dashed thick lines is the 95% prediction interval and the dashed thin lines are the estimated 95% bootstrap CI (the 2.5th and 97.5th percentiles for each time point). The light grey lines are the best fit of the function to 100 randomly selected bootstrap samples (out of the 500). The symbols show individual measurements (blue (circles), CN; orange (squares), MCI; red (asterisks), ADem). The vertical dashed and thick dotted lines represent the average expected time from ADem onset of individuals that are CN (27.75 years) and MCI (9.63 years) at baseline, respectively

For all markers, it is observed that there is a great deal of overlap in measurements across the different diagnostic states, i.e. no single marker is able to accurately distinguish between clinical states (Fig. 3—the parameter estimates of all the biomarker trajectory models are provided in Additional file 1: Table S2). Although it is difficult to draw any conclusions at the individual level, at the population level, the overall biomarker changes occurred during disease development are in agreement with those reported in previous studies; CSF Aβ_1–42_ levels are reduced, p-tau and t-tau levels in CSF and amyloid levels in the brain are elevated, and brain atrophy and hypometabolism, as well as cognitive and memory dysfunction, are observed.

**Fig. 4:**
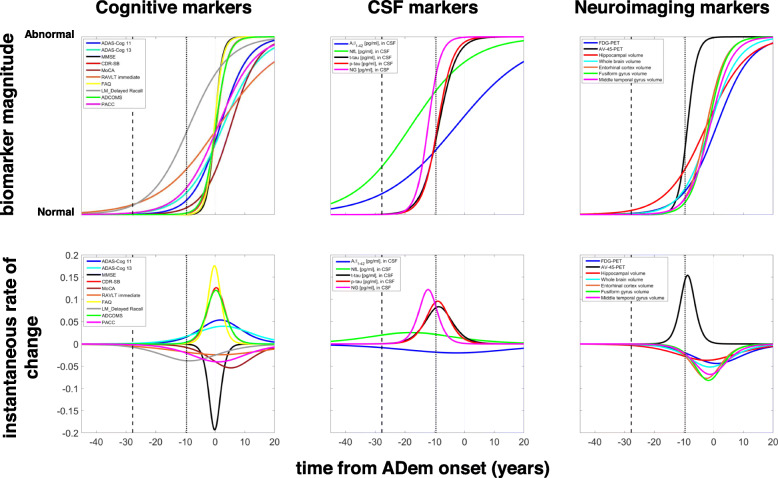
Normalised trajectories and rates of change. Top row: normalised (min-max) trajectories of cognitive, CSF and neuroimaging markers, where ‘normal’ is the value in healthy CN individuals (as *t*_ADem_ →  − ∞) and ‘abnormal’ is the value in severe ADem individuals (as *t*_ADem_ → ∞). Bottom row: rate of change of the (normalised) values of cognitive, CSF and neuroimaging markers. The vertical dashed and thick dotted lines represent the average expected time from ADem onset of individuals that are CN (27.75 years) and MCI (9.63 years) at baseline, respectively

**Fig. 5:**
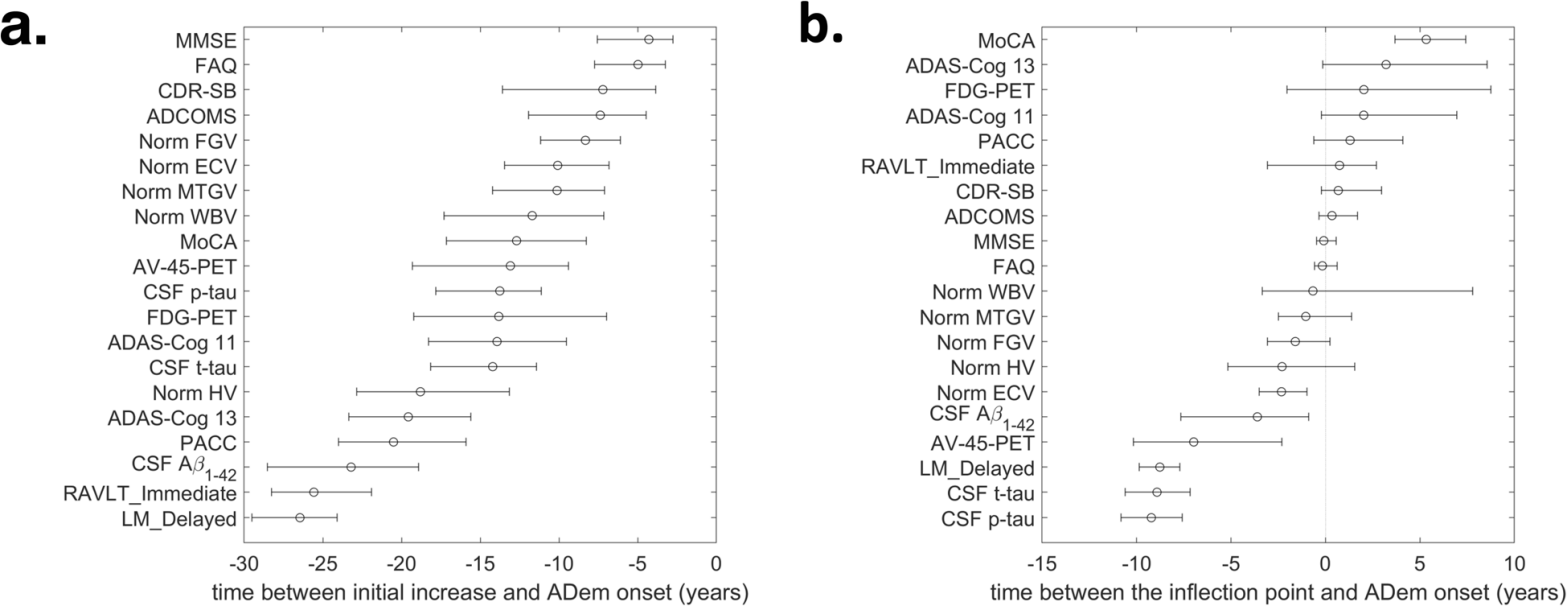
Initial biomarker changes and inflection points. **a** The estimated time of the initial change of each of the cognitive, CSF and neuroimaging markers. **b** The time at which the inflection point is reached. Error bars show the 95% CI for the mean, estimated by calculating the 2.5th and 97.5th percentiles of the outputs obtained from 500 bootstrap samples. Only the visits at which CSF Aβ_1–42_ was measured have been included in the analysis. The plasma markers, PiB-PET and CSF NfL and Ng, have been excluded. Norm HV, normalised hippocampal volume; Norm ECV, normalised entorhinal cortex volume; Norm FGV, normalised fusiform gyrus volume; Norm MTGV, normalised middle temporal gyrus volume; Norm WBV, normalised whole brain volume

Due to the high variability in measurements, a definite sequence of biomarker changes is difficult to clearly define. However, our model suggests that on average, the concentration of Aβ_1–42_ in CSF demonstrates slow pathologic changes occurring about 23.2 (95% CI, 18.9–28.6) years prior to the clinical presentation of ADem (Figs. 3, 4 and 5). The rate of change peaks much later, approximately 3.6 (95% CI, 0.9–7.7) years before the ADem clinical symptoms onset, and is followed by a slower deposition which tends towards a plateau. Detectable changes in CSF t-tau and p-tau proteins occur later displaying a similar dynamic, approximately 14 years prior to the ADem onset. No significant difference in the time of occurrence was observed (13.8 (95% CI, 11.1–17.8) years for p-tau and 14.2 (95% CI, 11.4, 18.2) years for t-tau). On average, PET evidence of Aβ accumulation (AV-45-PET) and brain hypometabolism on FDG-PET are also observed around the same time (Fig. 5).

The hippocampus tends to be the first brain structure affected by the disease; we estimate that detectable hippocampal atrophy could occur as early as the first signs of amyloid deposition (18.8 (95% CI, 13.1–22.8) years before the ADem onset). Changes in other structural neuroimaging markers, including the whole brain volume (11.7 (95% CI, 7.1–17.3) years before the ADem onset), are likely to occur much later in the course of the disease (Fig. 5).

Cognitive scores may better reflect continuous changes occurring during clinical progression, albeit with high variability (Fig. 3), with notable changes being observed closer to the onset of ADem. Memory dysfunction, indicated by detectable changes in the LM_Delayed and RAVLT_Immediate scores, could be observed even earlier than changes in CSF Aβ_1–42_, 26.5 (95% CI, 24.1–29.5) and 25.6 (95% CI, 21.9–28.3) years, respectively, before the onset of ADem clinical symptoms (Fig. 5 and Additional file 1: Section S6, Fig. S4). It is also observed that these markers worsen almost in parallel with this protein throughout the full progression period of the disease (Fig. 4). Changes in sensitive scores of clinical performance, such as PACC, could occur as early as 20.5 (95% CI, 15.9–24) years before ADem and could thus serve as indicators of early cognitive decline in preclinical AD populations. Global cognitive decline, as assessed by CDR-SB and MMSE, and functional abilities, as assessed by FAQ score, occur last, a few years before the ADem onset (7.2 (95% CI, 3.9–13.6), 4.3 (2.8–7.6) and 5 (3.2–7.74) years before ADem, respectively, see also Additional file 1: Section S6, Fig. S4 on the whole dataset). However, FAQ changes at a very high rate, with the highest acceleration occurring within 1 year after the ADem onset (0.16 (95% CI, 0.58–0.63) years after the onset).

Variations in the levels of plasma markers are difficult to discern. Changes in plasma NfL are more pronounced compared to that of the other plasma markers, with the NfL concentration rising at early stages in disease development. Elevated levels of plasma t-tau are observed at more severe clinical states [52].

Figure 5b illustrates the time between the inflection point and the ADem onset. This is a good measure in the sense that it can be used to order important changes in the levels of the markers. On average, the highest acceleration of decline in all functional and cognitive abilities, as well as FDG-PET hypometabolism, is expected to take place soon after the clinical manifestation of ADem, following cerebral atrophy, as assessed by the MRI brain volumetric measures. CSF markers, memory impairment (as assessed by LM_Delayed score) and AV-45 PET, which is one of the most direct measures of Aβ accumulation in the brain, could exhibit the highest rate of change much before the ADem onset [6, 13] (Fig. 5 and Additional file 1: Section S6, Fig. S4).

## Discussion

In this study, we aimed to characterise the continuous pathophysiological changes that may be observed across the clinical spectrum of AD and facilitate the interpretation of such changes in clinical settings. We first developed a mathematical model with a probabilistic structure to estimate the expected time interval for each individual from the time of measurement to the onset of a particular clinical state, focusing on the onset of ADem. We then employed these estimates to align short-term individual measurements of cognitive and biological markers on a clinical progression timeline and model their long-term temporal dynamics at the population level. It should be emphasised that although age is one of the important factors affecting the time to a clinical state, individuals are not aligned based on their age, and, despite the high variability, the overall biomarker trajectory can show changes that can occur across the clinical progression to ADem, independently of ageing.

It was shown that the probability of moving to a more severe clinical state increases almost exponentially with age. Depending on the age and clinical state, both the presence of an APOE ε4 allele and lower levels of education increase the likelihood and rate of developing symptoms of ADem, but the presence of APOE ε4 was shown to be the dominant factor (it is worth noting that studies have shown that APOE ε4 is linked to CSF Aβ_1–42_ accumulation [8, 53]). However, since ADNI is a sample of highly educated people (mean 16 years), the actual influence of education on the clinical expression of AD is unclear in this study and should be interpreted with caution. Many previous studies have shown an association between ADem onset and educational attainment, which could be partly attributed to resistance to cognitive decline due to cognitive reserve, literacy abilities, socioeconomic status, engagement in cognitively demanding work, social activities or occupational roles. This association could also be partly due to a bias in detecting ADem symptoms [54]. However, there are divergent views amongst researchers on the actual impact of education on the development of AD and the rate of progression [20, 55, 56].

The results support and contribute to the current understanding of the quantitative characteristics of the clinical progression of AD based on multiple clinical and biological measures [2, 26]. The timespan and sequence of biomarker changes are also in general agreement with observations in autosomal dominant AD [6, 57]. In this study, we estimated and reported the whole time span between the first detectable dynamic biomarker changes and the onset of ADem clinical symptoms. On average, first signs of memory dysfunction and amyloid deposition, as reflected by the levels of Aβ_1–42_ in CSF, could be detected at the pre-symptomatic stage up to about three decades before the occurrence of substantial cognitive and functional decline, which are mainly observed at early stages of ADem [2]. Both markers also seem to change at a similar rate during the disease progression. This may imply a link between memory deficits and elevated amyloid deposition, a view that is supported by previous reports [2, 58–61]. The value of episodic memory tests, and associations of changes in the performance in delayed recall tasks and the clinical progression to ADem, has also been observed and discussed in previous studies [2, 9, 10, 62–65]. However, it should be noted that such markers may not be sufficient for the improvement of participant recruitment and endpoint choice in very early-stage prevention trials. Even if first CSF Aβ_1–42_ changes and disease-associated subtle memory deficits could be detected very early at the preclinical stage, the short time window within which substantial changes occur and the fact that individuals can have amyloid pathology for a long period without development of substantial cognitive dysfunction and brain pathology, as shown in several preclinical studies [8, 61], demonstrate the difficulties of recruiting individuals at the preclinical stage of AD and present obvious challenges in the design of clinical trials of preventative treatments.

An interesting result derived from our analyses is the suggestion that changes in CSF NfL [40], a biomarker of neuronal damage, could also occur approximately over the same timescale as memory decline and Αβ_1–42_ accumulation in CSF. However, more data is required to support this suggested pattern. Progressing towards ADem, signs of memory dysfunction and changes in CSF Aβ_1–42_ are closely followed by first signs of cognitive decline, as assessed by sensitive cognitive performance measures (e.g. PACC), and alterations in brain structure measured by hippocampal atrophy. The value of such markers, and in particular the ability of Aβ_1–42_ in CSF, cognitive (free recall) and normalised hippocampal volume to predict the onset of symptomatic sporadic AD (time to CDR > 0), has also been reported in a recent study [8]. Estimated changes in other CSF measures such as t-tau and p-tau, brain structural measures and brain glucose metabolism are observed later. Loss of functional abilities occurs last, closer to the ADem onset. Significant atrophy in brain regions, such as the entorhinal cortex, fusiform gyrus and middle temporal gyrus, occurs after initial hippocampal atrophy. However, at later stages, their expected atrophy rate becomes higher than that of the hippocampus and whole brain, and the estimates of the time point at which the maximum rate occurs are relatively precise, which may be attributed to the smaller size of these structures. Hence, such brain regions may be more sensitive biomarkers to changes than bigger brain regions [66]. Significant volume loss in all brain regions is likely to be observed just before the clinical diagnosis of ADem and rapid decline in cognitive abilities, which may indicate an association.

The accurate measurement of proteins in the blood and identification of a clear association with brain pathology are still challenging, potentially due to the effect of factors that are not directly related to the disease stage of individuals, including clearance mechanisms and high levels of these proteins produced in the peripheral organs. However, the ease and low cost of measurements have created much interest in identifying reliable and sensitive peripheral blood-based markers that could serve as biomarkers of AD [67]. The changes in the plasma levels of proteins considered in this study were not as evident as those of other markers measured in CSF and the brain. However, plasma NfL seems to be a promising marker that can indicate pathological changes at early stages in disease development, and therefore, although not specific for AD, it could potentially be considered as a biomarker for the screening of related neurodegeneration and disease prognosis, monitoring and assessment [68–71].

The rate of change and sensitivity of the various markers varies across the different stages of the AD continuum [26], and so, the order in which they reach specific degrees of abnormality may differ. This, perhaps, suggests that the appropriateness of each marker for disease diagnosis and monitoring and use in clinical trials should be based on the disease stage [8, 72]. Considering the rate of change of potential markers instead of just a single absolute measure at a specific point in time may help identify the stage in disease progression [8]. For some markers, such as t-tau and p-tau, the results suggest that the average rate of change tends to zero around the occurrence of clinical ADem symptoms. This may be an implication of severe and irreversible pathological changes that may happen well before individuals reach severe clinical stages. On the other hand, some biomarkers, such as neuroimaging markers, remain dynamic even after onset, which may imply that there might be late stages where therapeutic interventions can be effective. Such late biomarkers may also be more useful in monitoring disease progression at severe disease stages [13]. In this study, it is shown that substantial dynamic changes in Aβ_1–42_ are also expected to continue to occur after the clinical presentation of ADem. This contradicts previous studies in preclinical Alzheimer’s disease which suggested that Aβ_1–42_ reaches a plateau by the time of ADem onset [61, 72]. However, the late disease stages are not well represented in the ADNI sample which may have biassed the estimation of the late biomarker behaviour. Thus, whether biomarkers reach a plateau or continue to decelerate after ADem onset needs to be validated when more longitudinal data at late stages become available.

In addition to modelling the biomarker trajectories using all available ADNI data, the trajectories have also been estimated in a sample of individuals that may be more likely to be in the Alzheimer’s continuum due to the evidence of brain Aβ accumulation observed during the study [16]. It has been shown that, on average, individuals in the amyloid-positive group tend to move faster towards more severe clinical states, potentially because they are at an advanced stage in disease development. However, no remarkable differences were observed in the average qualitative and quantitative behaviour of biomarkers throughout the disease progression when compared to the biomarkers estimated in the entire sample, especially in the late disease stages. This may be attributed to the fact that ADNI participants are a highly selective group who have an increased risk of developing the disease (almost 50% of the individuals are classified in the amyloid-positive group). Differences, however, may be pronounced when comparing the trajectories during follow-up in the study between the groups of those that have and those that have not shown elevated levels of Aβ at baseline [37].

It is worth emphasising that whether biomarker changes as predicted by the models can be detected in a clinical study is uncertain. The high variability and overlap between the measurements at the different clinical states confirm difficulties in detecting significant biomarker changes and in making accurate predictions at the individual level. Hence, the order at which changes in biomarkers can be detected might be different from the current general view of biomarker change patterns [12]. In addition, due to the lack of knowledge of the exact impact of each biomarker change on the biological process of disease development, it is difficult to assess their importance based on the changes observed, as a small change in one marker might be more important than a large change in another marker.

The model estimations are contingent on the validity of a series of simple assumptions. The mathematical model assumes that at every given state, the future of the system (for example, the probability of progressing to ADem in the next year given the current state is MCI) is entirely independent of the history of the system (for example, at which states individuals were before and for how long). However, this limitation is partly mitigated by the fact that transition probabilities in the model depend on the current diagnostic state and age, and thus, depending on the clinical state, older individuals are more likely to transition to more severe states independently of the system’s history. In addition, the model relies on pre-defined distinct clinical states and the accuracy of clinical diagnosis. Despite the high measurement noise in clinical scores and the high rate of clinical misdiagnosis [22], it was also assumed that individuals transitioned to a different clinical state at the visit when the diagnostic criteria were satisfied, even though, due to the long-term/gradual nature of AD progression and the sequential change in different pathophysiological processes, defining any other distinct boundaries between the states is challenging. The current categorisation of individuals in clinical groups is also based on a simplified set of criteria, which does not fully represent their biomarker profile and does not take into account various sources of uncertainty. Hence, although this classification is useful in clinical practice, it increases heterogeneity within and overlap between the biomarker measurements in the clinical groups. Indeed, the overlap for some biomarkers could also be partly attributed to neurobiological changes occurring in non-Alzheimer’s ageing population, due for example to ageing or the presence of other pathological processes and neurodegenerative or cardiovascular diseases, that can be present in all clinical stages [73].

As a biomarker model, and based on the current understanding of the pathophysiological processes that are associated with AD [26], a sigmoid function of time was chosen to describe biomarker temporal dynamics. Despite the high variability and the limited data to support very early and late stages in disease progression, this choice was supported by the available data and it described quite well the dynamic behaviour of most of the biomarkers. It also allowed us to estimate when significant biomarker changes occur, as well as the point at which biomarkers are most likely to reach the maximum rate of change before they potentially begin to plateau. In addition, it was assumed that all individuals will follow the same pathway of neuropathological changes towards ADem. Although the sample of individuals considered are at high risk of developing ADem, this may have resulted in the overestimation of the expected times to a clinical state, which may have in turn influenced the estimation of the biomarker dynamics and the point at which first significant changes occur.

Although all biomarker data has been synchronised on the same disease progression timeline independently of the levels of any of the biomarkers, estimating the trajectories utilising a complete dataset for all biomarkers would enable us to make a better comparison between the biomarker dynamics, and in particular the point at which the first significant change and the inflection point occur. Due to the limited data, a complete dataset could not support the model of changes in many biomarkers and the estimation of the quantities considered. When utilising the whole dataset, the results produced were more precise for biomarkers with more measurements, even though in this case many biomarkers may not be comparable. However, in any case, a definite sequence of pathophysiological events that lead to ADem cannot yet be suggested given the available data. More frequent measurements taken systematically at the same time for all biomarkers would enable us to reduce data variability and produce more precise results. Increasing the sample size, reducing data heterogeneity and defining the error of each measurement/assay would greatly facilitate the detection of even earlier changes than the ones presented in the current study.

The model can be used to estimate the biomarker trajectories in different risk groups. It can also be extended to incorporate other potential confounding variables, such as the level of specific biomarkers; so, given the characteristics of an individual and the levels of biomarkers included in the model, the expected time to ADem, or other disease states, will be estimated. The same methodology can also be applied to describe the sequence and magnitude of biomarker changes with respect to the time required to reach a specific biomarker value, e.g. a defined biomarker threshold/cut-off point [8, 74]. The estimation of these times could help in the identification of individuals at the earliest disease stages that could be recruited in secondary prevention trials, where, currently, participants are included if they have already developed a biomarker pathology defined by conventional biomarker thresholds.

## Conclusions

The model presented here estimates the continuous changes that can be observed in the level of cognitive and biological markers during the clinical progression of cognitively unimpaired individuals to Alzheimer’s clinical syndrome. Despite the high variability within and between individuals at the different clinical states, it demonstrates the long period of asynchronous pathologic changes occurring along with the clinical progression and supports and complements previous conceptual and hypothetical models on the pathophysiological cascade [26]; amyloid deposition and signs of memory decline can occur in cognitively unimpaired individuals as early as 30 years prior to the onset of Alzheimer’s clinical syndrome. A sequence of events, including tauopathy, structural brain changes and neurodegeneration, follows before the onset of severe cognitive symptoms. Such models can provide insights into the underlying causes of the clinical symptoms and support the choice of appropriate endpoints in clinical trials according to the stage in disease progression. They can also facilitate the identification of the position of individuals in the pathophysiological continuum, which will be an important tool for improving the recruitment process in clinical trials and the assessment of treatment outcomes.

## Supplementary information

**Additional file 1:** Supplementary information, tables and figures.

## Data Availability

The datasets generated and analysed during the current study are available in the ADNI data repository, http://adni.loni.usc.edu/data-samples/access-data/.

## Abbreviations

Aβ: Amyloid-β
ADAS-Cog: Alzheimer’s Disease Assessment Scale-Cognitive Subscale
ADCOMS: Alzheimer’s Disease Composite Score
ADNI: Alzheimer’s Disease Neuroimaging Initiative
APOE: Apolipoprotein E
CDR-SB: Clinical Dementia Rating Scale-Sum of Boxes
CN: Cognitively normal
F18-AV-45: Florbetapir
FAQ: Functional Activities Questionnaire
FDG: ^18^F-fluorodeoxyglucose
ICV: Intracranial volume
MCI: Mild cognitive impairment
MMSE: Mini-Mental State Examination
MoCA: Montreal Cognitive Assessment
NfL: Neurofilament light
Ng: Neurogranin
PACC: Preclinical Alzheimer Cognitive Composite
PiB: Pittsburgh compound B
p-tau: Phosphorylated tau
RAVLT: Rey’s Auditory Verbal Learning Test
SUVr: Standardised uptake value ratio
t-tau: Total tau
LM_Delayed: Logical Memory-Delayed recall
