# Supplementary material for: The dynamics of biomarkers across the clinical spectrum of Alzheimer’s disease
Source: Alzheimers Res Ther. 2020 Jun 13;12:74. doi: 10.1186/s13195-020-00636-z (PMC7293779; doi:10.1186/s13195-020-00636-z)
Supplement: Supplementary file 1 — Additional file 1: Supplementary information, tables and figures. [file 13195_2020_636_MOESM1_ESM.pdf]

Supplementary Information

The dynamics of biomarkers across the clinical spectrum of Alzheimer's disease

Christoforos Hadjichrysanthou, Stephanie Evans, Sumali Bajaj, Loizos C. Siakallis, Kevin McRae-McKee, Frank de Wolf, Roy M. Anderson, for the Alzheimer's Disease Neuroimaging Initiative

S.1 Additional details on the statistical model

A GLMM with fixed effects of *m* variables and a random intercept term takes the general form

$$ln\left(\frac{p_{A_{ij},B_{ij}}}{1-p_{A_{ij},B_{ij}}}\right) = \beta_0 + \sum_{k=1}^{m} \beta_k \; x_{\kappa ij} + \; b_i \; + \varepsilon_{ij}, \tag{S1.1}$$

where $p_{A_{ij},B_{ij}}$ is the probability of participant i transitioning from state A to state B at observation j. β_0 is the mean intercept and β_k is the log odds ratio associated with a one unit increase in variable x_k. $b_i \sim N(0, \sigma_b^2)$ represents a specific deviance from β_0 for individual i which accounts for the variability in the likelihood of transitioning between individuals. $\varepsilon_{ij} \sim N(0, \sigma_\varepsilon^2)$ represents the random error which accounts for the variability within individuals. Hence, parameters σ_b^2 and σ_ε^2 represent the variance between and within individuals, respectively.

S.2 Derivation of the expected time to MCI and ADem

The process described in the schematic diagram of Fig. 1 can be represented by the following 3×3 transition matrix:

$$
{}^{a}T^{\{S\}} = \begin{array}{c} \\ \text{CN} \\ \text{MCI} \\ \text{ADem} \end{array} \begin{array}{c} \begin{array}{ccc} \text{CN} & \text{MCI} & \text{ADem} \end{array} \\ \begin{bmatrix} {}^{a}p^{\{S\}}_{CN,CN} & {}^{a}p^{\{S\}}_{CN,MCI} & 0 \\ {}^{a}p^{\{S\}}_{MCI,CN} & {}^{a}p^{\{S\}}_{MCI,MCI} & {}^{a}p^{\{S\}}_{MCI,ADem} \\ 0 & 0 & 1 \end{bmatrix} \end{array},
$$

where ${}^{a}p^{\{S\}}_{A,B}$ is the probability of an individual at age a and with a set of time-invariant characteristics {S} transitioning from state A to state B within one year. Let ${}^{a}E^{\{S\}}_{A,B}$ be the expected number of steps (years) required for the chain to reach state B for the first time given that it starts at age a and state A.

${}^{a}E^{\{S\}}_{CN,MCI}$ is given by the solution of the following system:

$$
{}^{a}E^{\{S\}}_{CN,MCI} = {}^{a}p^{\{S\}}_{CN,CN} \, {}^{a+1}E^{\{S\}}_{CN,MCI} + 1. \tag{S2.1}
$$

Solving equation (S2.1) inductively we get

$$
{}^{a}E^{\{S\}}_{CN,MCI} = \sum_{m=a-1}^{\infty} \prod_{l=a}^{m} {}^{l}p^{\{S\}}_{CN,CN}. \tag{S2.2}
$$

Similarly, ${}^{a}E^{\{S\}}_{CN,ADem}$ and ${}^{a}E^{\{S\}}_{MCI,ADem}$ are given by the solution of the following system:

$$
{}^{a}E^{\{S\}}_{CN,ADem} = {}^{a}p^{\{S\}}_{CN,CN} \, {}^{a+1}E^{\{S\}}_{CN,ADem} + {}^{a}p^{\{S\}}_{CN,MCI} \, {}^{a+1}E^{\{S\}}_{MCI,ADem} + 1, \tag{S2.3}
$$

$$
{}^{a}E^{\{S\}}_{MCI,ADem} = {}^{a}p^{\{S\}}_{MCI,CN} \, {}^{a+1}E^{\{S\}}_{CN,ADem} + {}^{a}p^{\{S\}}_{MCI,MCI} \, {}^{a+1}E^{\{S\}}_{MCI,ADem} + 1. \tag{S2.4}
$$

The system (S2.3)-(S2.4) can be written in the following matrix form:

$$
\begin{bmatrix} {}^{a}E^{\{S\}}_{CN,ADem} \\ {}^{a}E^{\{S\}}_{MCI,ADem} \end{bmatrix} = \begin{bmatrix} {}^{a}p^{\{S\}}_{CN,CN} & {}^{a}p^{\{S\}}_{CN,MCI} \\ {}^{a}p^{\{S\}}_{MCI,CN} & {}^{a}p^{\{S\}}_{MCI,MCI} \end{bmatrix} \begin{bmatrix} {}^{a+1}E^{\{S\}}_{CN,ADem} \\ {}^{a+1}E^{\{S\}}_{MCI,ADem} \end{bmatrix} + \begin{bmatrix} 1 \\ 1 \end{bmatrix}. \tag{S2.5}
$$

Solving (S2.5) inductively we derive:

$$
\begin{bmatrix} {}^{a}E^{\{S\}}_{CN,ADem} \\ {}^{a}E^{\{S\}}_{MCI,ADem} \end{bmatrix} = \sum_{m=a-1}^{\infty} \prod_{l=a}^{m} \begin{bmatrix} {}^{l}p^{\{S\}}_{CN,CN} & {}^{l}p^{\{S\}}_{CN,MCI} \\ {}^{l}p^{\{S\}}_{MCI,CN} & {}^{l}p^{\{S\}}_{MCI,MCI} \end{bmatrix} \begin{bmatrix} 1 \\ 1 \end{bmatrix}. \tag{S2.6}
$$

S.3 Available data

Cognitive markers

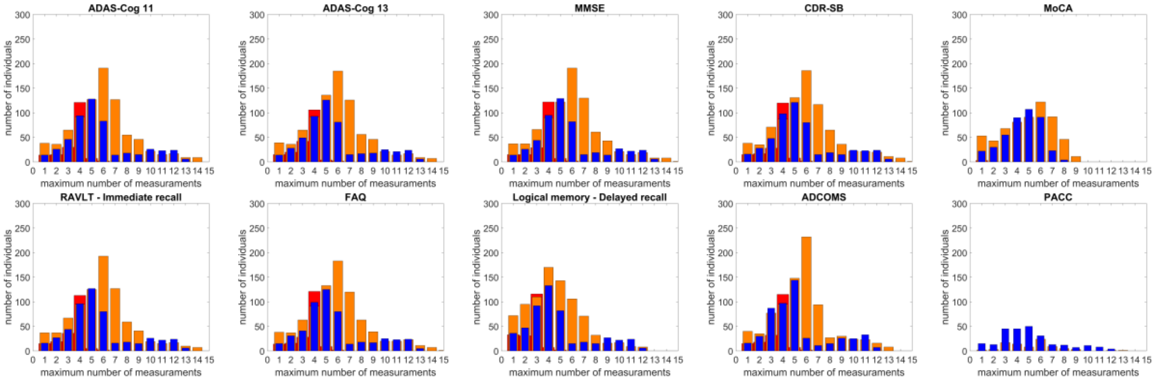

CSF markers

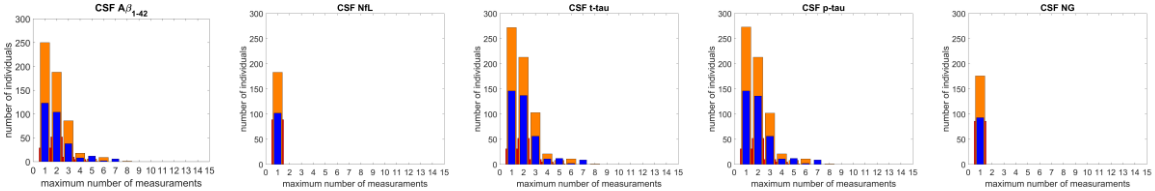

Plasma fluid markers

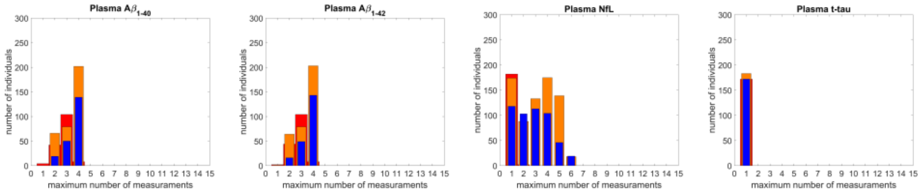

Neuroimaging markers

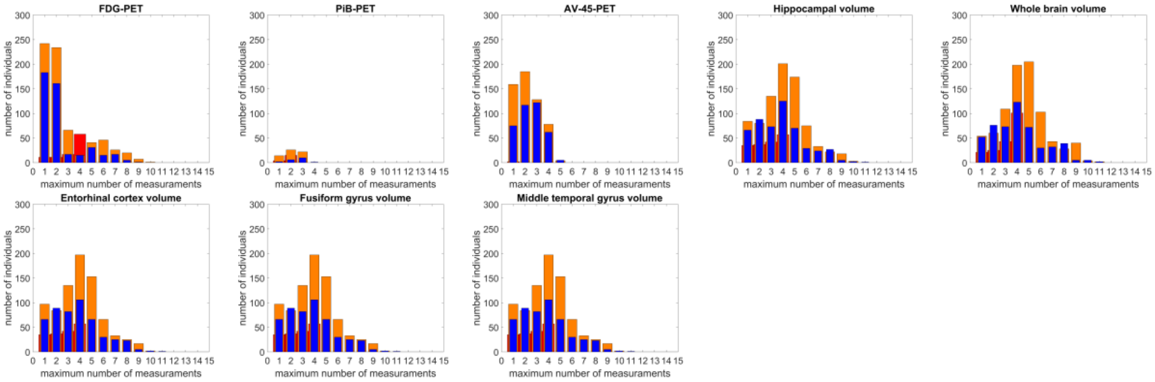

Fig. S1. Histograms showing the measurements used for the development of each marker trajectory in Fig. 3. Each bar shows the number of individuals that have exactly 1, 2, 3, etc. measurements, coloured by their clinical diagnosis at baseline; blue: CN, orange: MCI, red: ADem.

Table S1. Total number of measurements of each marker at the visits where CSF Aβ$_{1-42}$ was measured. These were utilised for the estimation of the first significant biomarker change and the inflection point in Fig. 5.

	Number of measurements		
	**CN**	**MCI**	**ADem**
**Cognitive markers**			
ADAS-Cog 11	575	888	355
ADAS-Cog 13	573	883	347
MMSE	575	890	358
CDR-SB	571	887	358
MoCA	352	551	86
RAVLT_Immediate	574	886	348
LM_Delayed	573	887	351
FAQ	570	886	357
ADCOMS	571	884	355
PACC	575	890	358
**CSF markers**			
Aβ$_{1-42}$	575	891	358
t-tau	569	890	356
p-tau	567	889	356
NfL*	82	160	85
Ng*	73	153	82
**Neuroimaging markers**			
FDG-PET	361	641	172
AV-45-PET	341	533	81
Hippocampus	460	714	242
Whole brain	494	820	302
Entorhinal cortex	446	698	234
Fusiform gyrus	446	698	234
Middle temporal gyrus	446	698	234
PiB*	15	36	22

* Due to the limited data the model could not be supported for these markers.

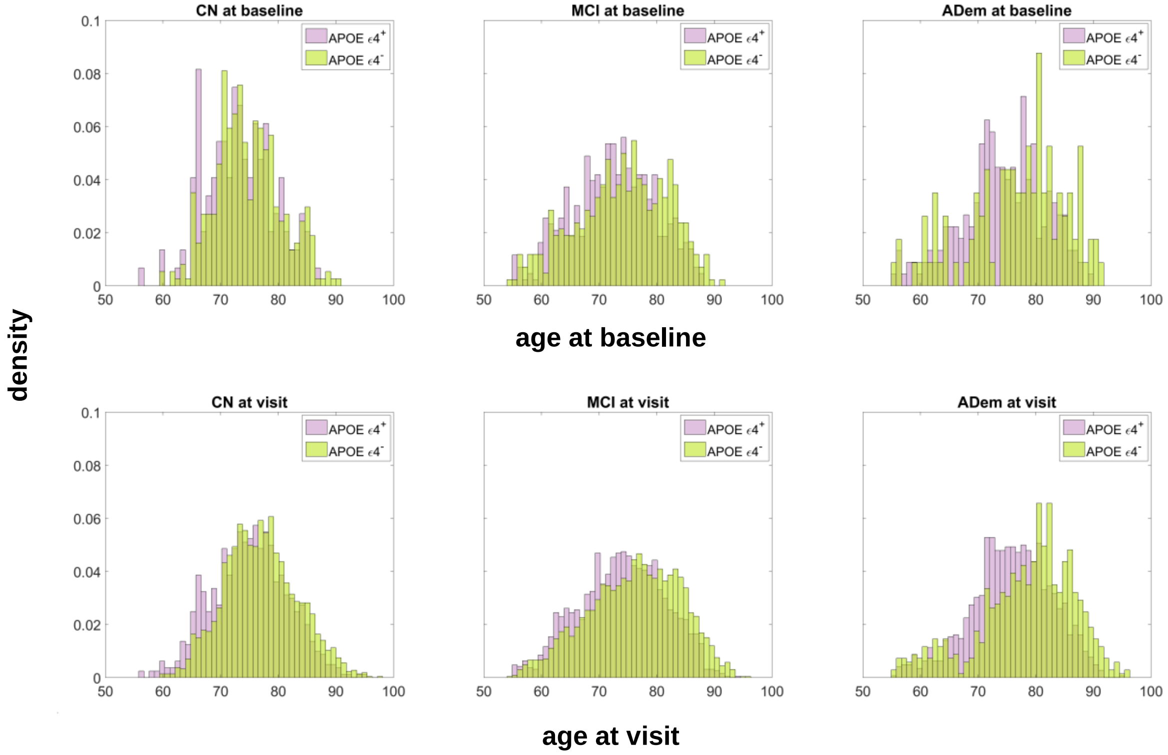

Fig. S2. Age distributions. APOE ε4 positive and negative groups at baseline (top row) and at any visit (bottom row).

S.4 Transition probabilities and expected times to a more severe state – Effect of educational attainment

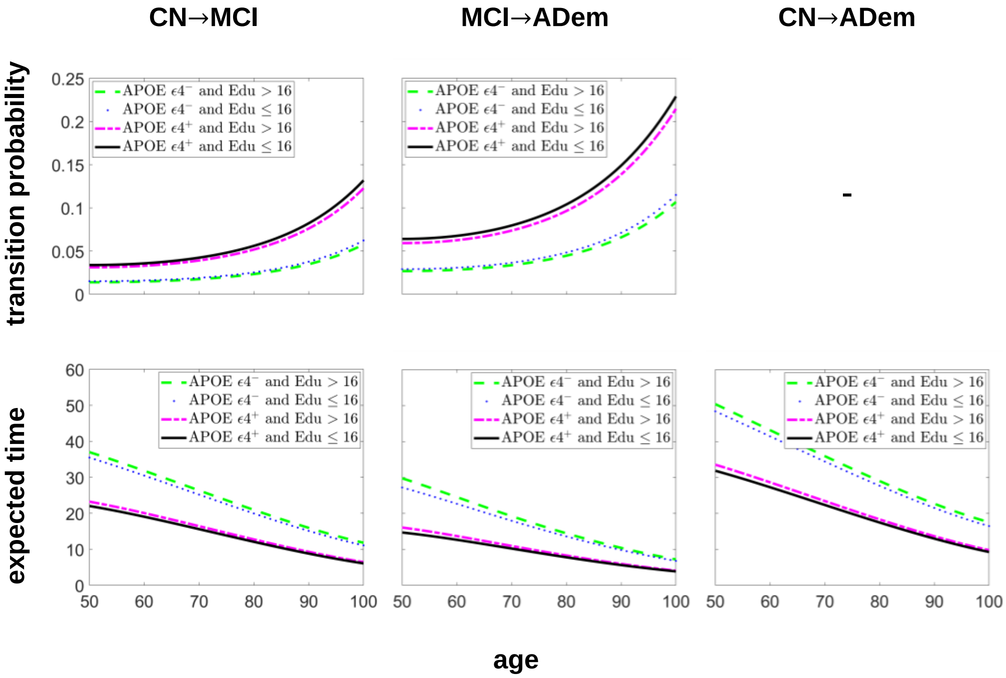

Fig. S3. Probabilities and times to a more severe clinical state: the effect of educational attainment. Mean probabilities of transitioning to a more severe state within one year (top), and the expected time to reach a more severe state (bottom), as a function of age in different risk groups defined by the educational level and APOE ε4 status. It is observed that higher educational attainment tends to decrease the mean of the probability of developing ADem and slow down the rate of clinical progression, but more data from individuals from a broad range of educational backgrounds is required to support this result. Edu = Education.

S.5 Best fits

Table S2. Best-fit for each marker: mean parameter values obtained from the model and 95% confidence interval (in brackets) computed using 500 bootstrap samples.

	Parameter estimates			
	r_1	r_2	r_3	r_4
**Cognitive markers**				
ADAS-Cog 11	6.36 (6.05, 6.82)	34.03 (30.19, 39.47)	-0.23 (-0.30, -0.18)	2.04 (0.76, 3.82)
ADAS-Cog 13	9.22 (8.80, 9.78)	51.08 (46.49, 57.90)	-0.17 (-0.20, -0.15)	2.18 (0.77, 4.01)
MMSE	28.52 (28.44, 28.63)	19.17 (18.11, 20.00)	-0.54 (-0.73, -0.38)	0.29 (-0.10, 0.82)
CDR-SB	0.71 (0.68, 0.74)	7.16 (6.79, 7.63)	-0.67 (-0.81, -0.52)	0.03 (-0.18, 0.31)
MoCA	25.65 (25.38, 25.88)	0.00 (0.00, 0.00)	-0.19 (-0.22, -0.16)	5.53 (4.73, 6.34)
RAVLT_Immediate	47.67 (45.84, 49.15)	2.15 (0.00, 7.71)	-0.10 (-0.12, -0.09)	-0.01 (-2.34, 1.36)
LM_Delayed	14.26 (13.94, 14.55)	0.00 (0.00, 0.00)	-0.17 (-0.18, -0.15)	-8.07 (-8.70, -7.57)
FAQ	1.33 (1.25, 1.42)	19.22 (18.56, 19.98)	-0.78 (-0.94, -0.66)	-0.31 (-0.45, -0.16)
ADCOMS	0.11 (0.10, 0.11)	0.89 (0.84, 0.96)	-0.55 (-0.68, -0.42)	0.01 (-0.22, 0.41)
PACC	0.23 (-0.11, 0.51)	-29.95 (-33.90, -26.95)	-0.16 (-0.17, -0.14)	2.34 (1.08, 3.89)
**CSF markers**				
$A\beta_{1\text{-}42}$	1205.05 (1097.10,1353.40)	249.65 (203.00, 474.21)	-0.09 (-0.16, -0.06)	-3.72 (-8.05, -0.79)
NfL	834.03 (405.00, 1101.20)	2009.77 (1562.10,3785.50)	-2.77 (-30.28, -0.03)	-12.63 (-31.65, 14.51)
t-tau	233.67 (226.49, 240.27)	366.02 (349.56, 387.11)	-0.35 (-0.55, -0.20)	-8.31 (-9.85, -6.40)
p-tau	21.36 (20.69, 21.98)	35.95 (34.29, 37.94)	-0.42 (-0.74, -0.22)	-8.79 (-10.29, -7.19)
Ng	325.40 (272.98, 375.88)	561.70 (512.32, 641.40)	-4.96 (-28.47, -0.12)	-11.37 (-15.86, -6.77)
**Plasma fluid markers**				
$A\beta_{1\text{-}40}$	163.75 (161.74, 166.10)	0.05 (0.00, 0.16)	-	-
$A\beta_{1\text{-}42}$	39.37 (38.90, 39.82)	0.00 (0.00, 0.00)	-	-
NfL	35.94 (28.33, 38.19)	68.04 (54.85, 180.51)	-0.86 (-4.33, -0.05)	-2.59 (-7.13, 36.68)
t-tau	3.10 (2.95, 3.26)	0.01 (0.00, 0.02)	-	-
**Neuroimaging markers**				
FDG-PET	1.31 (1.29, 1.32)	0.89 (0.71, 0.98)	-0.18 (-0.27, -0.12)	0.76 (-1.67, 5.80)
PiB-PET	1.45 (1.34, 1.68)	1.93 (1.81, 2.02)	-25.49 (-49.99, -0.41)	-14.27 (-67.24, -2.38)
AV-45-PET	1.10 (1.08, 1.11)	1.37 (1.34, 1.39)	-0.80 (-1.59, -0.36)	-9.19 (-10.15, -8.37)
Hippocampus	0.0049 (0.0049, 0.0050)	0.0029 (0.0026, 0.0033)	-0.16 (-0.20, -0.12)	-2.02 (-4.09, 0.52)
Whole brain	0.68 (0.678, 0.683)	0.60 (0.58, 0.60)	-0.27 (-0.35, -0.21)	-2.05 (-2.85, -1.05)
Entorhinal cortex	0.0025 (0.0025, 0.0025)	0.0015 (0.0014, 0.0016)	-0.26 (-0.32, -0.21)	-2.37 (-3.22, -1.35)
Fusiform gyrus	0.0119 (0.0118 0.0119)	0.0089 (0.0086 0.0092)	-0.30 (-0.37, -0.24)	-2.00 (-2.86 -1.00)
Middle temporal gyrus	0.0133 (0.0133, 0.0134)	0.0099 (0.0095, 0.0102)	-0.30 (-0.37, -0.24)	-1.84 (-2.69, -0.86)

S.6 Comparison of the times at which the first changes and the inflection points of the biomarker trajectories occur when using the whole dataset

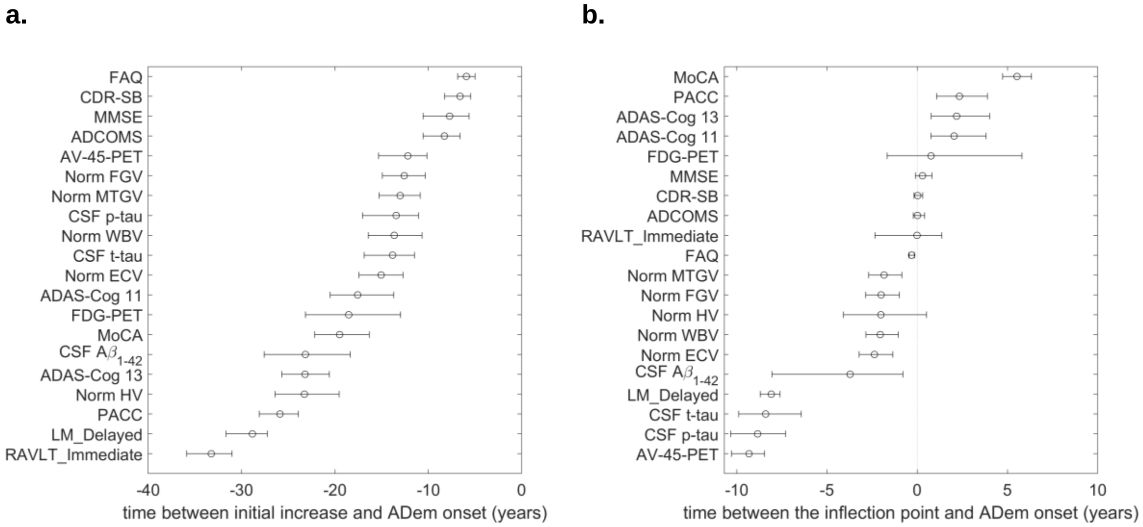

Fig. S4. Initial biomarker changes and inflection points. a Estimated time of initial change of each of the cognitive, CSF and neuroimaging markers. **b** Time at which the inflection point is reached. Error bars show the 95% confidence interval for the mean, estimated by calculating the 2.5th and 97.5th percentiles of the outputs obtained from 500 bootstrap samples. The whole dataset (Fig. S1) has been used for this analysis. The plasma markers, PiB-PET and CSF NfL and Ng have been excluded. Norm HV = normalised hippocampal volume; Norm ECV = normalised entorhinal cortex volume; Norm FGV = normalised fusiform gyrus volume; Norm MTGV = normalised middle temporal gyrus volume; Norm WBV = normalised whole brain volume.

S.7 Further model evaluation

In this section, we perform additional analysis to further evaluate the algorithms that have been developed for the estimation of the biomarker trajectories.

S.7.1 Validation of the sigmoidal and linear models

In addition to bootstrapping that has been performed to produce confidence intervals for the estimates of the average biomarker trajectories, we evaluated the performance of the sigmoidal and linear models using a 10-fold cross-validation technique. The sample used for the estimation of the expected times to the onset of ADem (Table 1), was randomly partitioned into 10 (non-overlapping) subsamples of approximately equal size of observations. We then performed 10-fold cross-validation and calculated the Normalised Root Mean Square Error for evaluation (NRMSE, normalised by the difference between the maximum and the minimum value in the testing dataset). The output of this experiment is shown in Fig. S5.

Cognitive markers

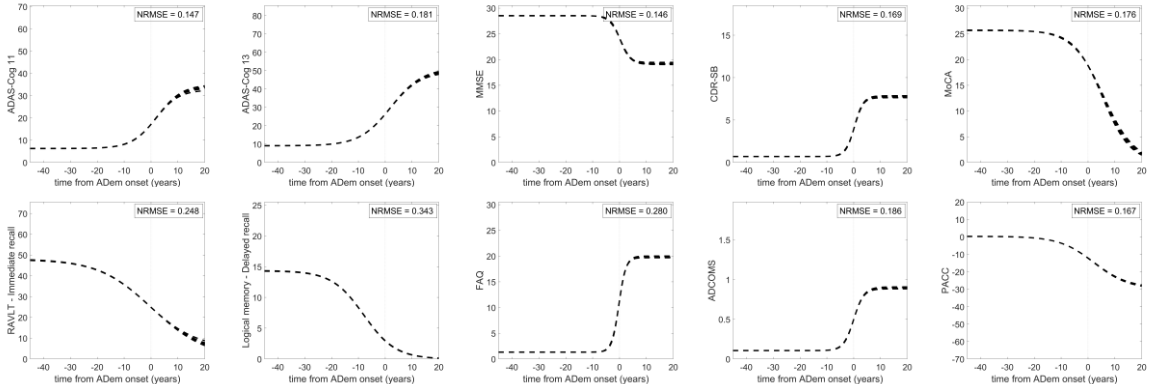

CSF markers

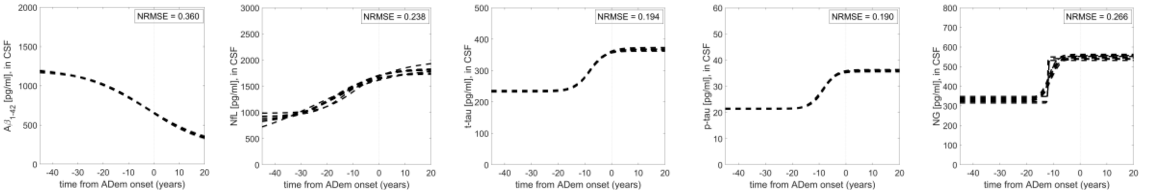

Plasma fluid markers

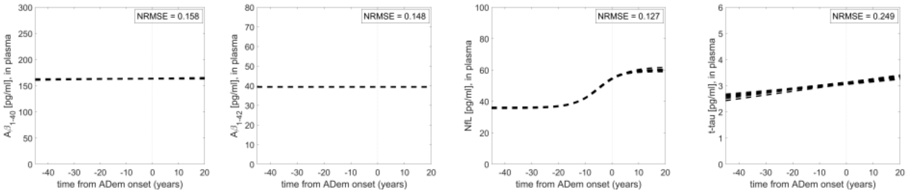

Neuroimaging markers

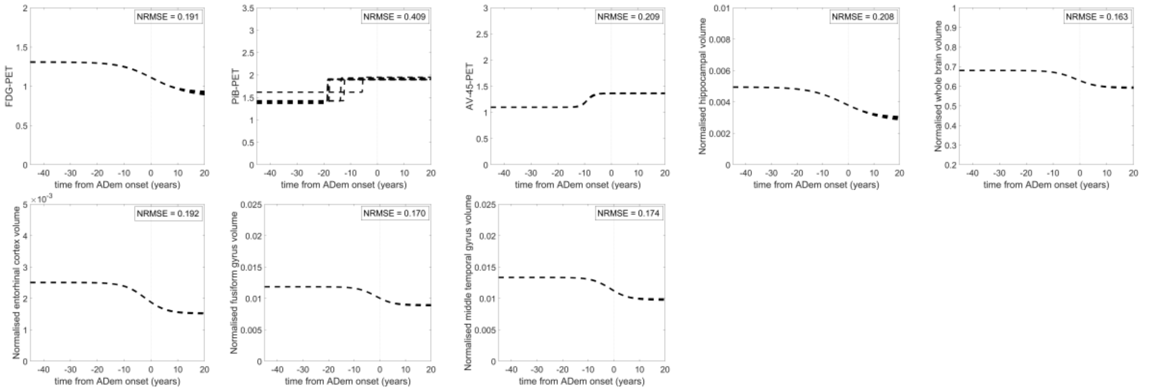

Fig. S5. Biomarker trajectories: 10-fold cross-validation of the sigmoidal and linear models. The fitting procedure is implemented ten times. Each time, nine of the ten subsets are used as the training set, and one subset is left out as the test set. The dashed lines show the best-fit for the training sets in each of the 10 rounds of the training-testing procedure. The model output in each round is evaluated using the Normalised Root Mean Square Error (NRMSE). Each figure shows the average NRMSE for the respective biomarker.

S.7.2 A holdout validation

We performed a single train-and-test experiment using the holdout validation method. The data (Table 1) was divided into two disjoint subsets. The first subset, the ‘test/hold-out’ set, includes all individuals that were CN at baseline and developed MCI and ADem during the longitudinal study. Hence, for each individual in the test set the approximate time of transition to each clinical state is known and their longitudinal biomarker data can be aligned according to the observed time between each measurement and the onset of ADem clinical symptoms. There are 21 such individuals whose characteristics are presented in Table S3. The model was trained on the remaining dataset (the ‘training’ set). The estimated biomarker trajectories were evaluated on the test set using the NRMSE (see Fig. S6).

Table S3. Number and characteristics of individuals that transitioned from CN to MCI and then to ADem during the study.

**Total number of CN individuals at baseline that developed MCI and ADem during the study**		21
**Chronological age at baseline (years)**	**≤70**	1
	**70<Age≤80**	18
	**>80**	2
**Gender**	**Women**	13
	**Men**	8
**Years of education**	**≤16**	14
	**>16**	7
**Genetic Background (APOE ε4)**	**Non-carriers of ε4 allele**	10
	**Carriers of 1 ε4 allele**	11
	**Carriers of 2 ε4 alleles**	0
**Other characteristics (standard deviation of the mean in brackets)**		
**Average age at baseline (years)**		75.37 (3.87)
**Average number of years of education**		15.76 (2.66)
**Average follow-up (years)**		8.16 (2.96)
**Mean time to ADem (years)**		6.60 (2.71)

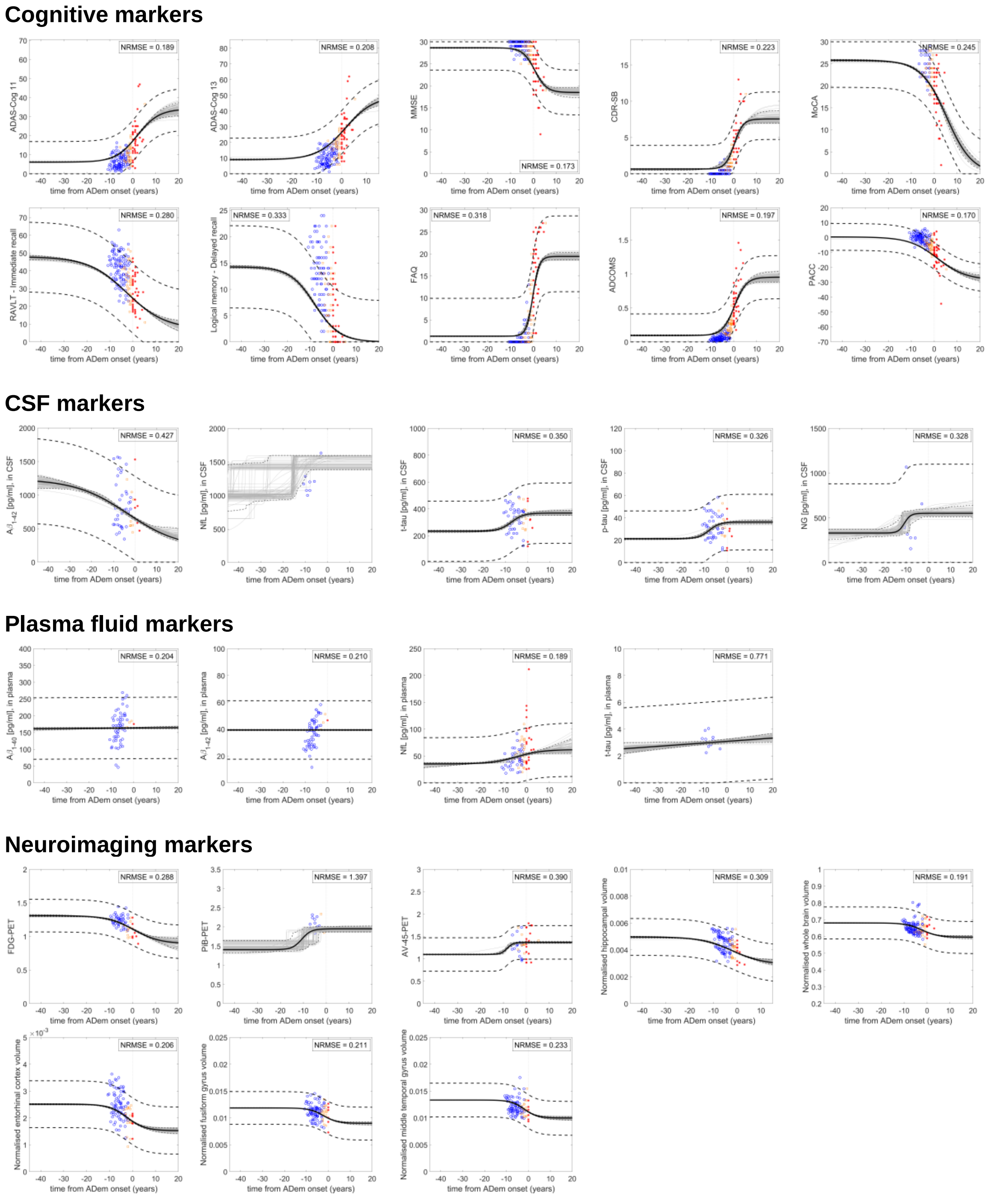

Fig. S6. Biomarker trajectories: holdout validation. The model is trained on the original sample (Table 1) excluding 21 individuals that entered the study as CN and developed MCI and ADem during the study (Table S3). The data from these individuals is used as the test set to evaluate the estimation of the biomarker trajectories. The black solid line represents the best fit of the sigmoid function (linear for $A\beta_{1-40}$, $A\beta_{1-42}$ and t-tau in plasma) for the training set. The dashed thick lines show the 95% prediction interval and the dashed thin lines the estimated 95% bootstrap confidence interval (the 2.5th and 97.5th percentiles for each time point). The light grey lines are the best fit of the function to 100 randomly selected bootstrap samples (out of the 500). The symbols show individual measurements in the test set (blue (circles): CN, orange (squares): MCI, red (asterisks): ADem). NRMSE: Normalised Root Mean Square Error.

S.8 Effect of amyloid positivity

964 (47.86%) individuals of the ADNI sample that we considered, are classified in the amyloid-positive group. At baseline, 230 (23.86%) of them are CN, 498 (51.66%) are MCI and 236 (24.48%) ADem. The proportion of APOE ε4 carriers in amyloid-positives is higher than that of non-carriers. In particular, from those for which APOE ε4 status is recorded, 570 (60.96%) are carriers of APOE ε4 (at baseline, 92 CN, 308 MCI and 170 ADem) and 365 (39.04%) non-carriers (at baseline, 119 CN, 185 MCI and 61 ADem). On average, the group of amyloid-positive individuals tends to have a higher chance of developing MCI and ADem than the whole group of individuals. Thus, the expected time to reach ADem is lower. The difference is more pronounced in the groups of non-carriers of the APOE ε4 genotype, whereas the difference in the group of APOE ε4 carriers is very small (Fig. S7), which may be due to the high proportion of carriers of APOE ε4 that are also amyloid-positive (71.25%). The average expected time to ADem of amyloid-positive individuals that have been classified as CN at baseline is 23.88 years and that of those that have been classified as MCI at baseline is 7.43 years (about four and two years, respectively, lower than those predicted for the whole sample). The output of the model that incorporates the effect of education suggests that the educational level has almost no influence on the chance of amyloid-positive individuals transitioning to a more severe clinical state within one year, and thus on the expected time to a clinical state (Fig. S8), independently of age. All the above yields the production of biomarker trajectories similar to those predicted in the whole sample (see Fig. S9, Table S4).

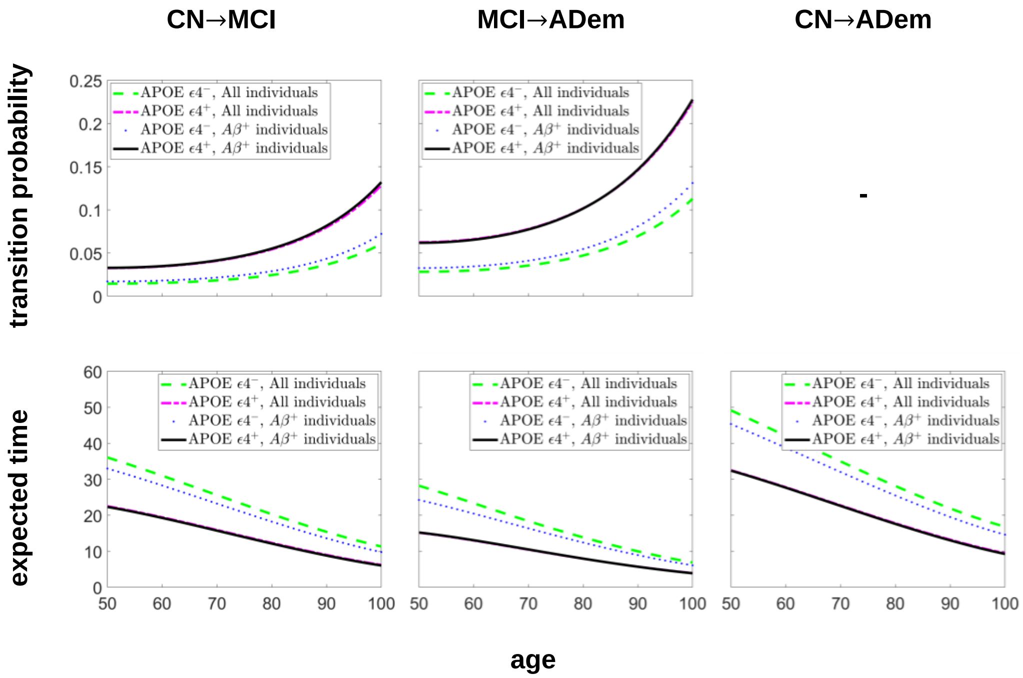

Fig. S7. Probabilities and times to a more severe clinical state: the effect of amyloid positivity. Mean probabilities of transitioning to a more severe state within one year (top), and the expected time to reach a more severe state (bottom), as a function of age in amyloid-positive individuals. For comparison, the transition probabilities and expected times in all individuals, irrespective of the Aβ status, are also presented. In both cases, the probabilities and times have been calculated in the groups of carries and non-carriers of APOE ε4.

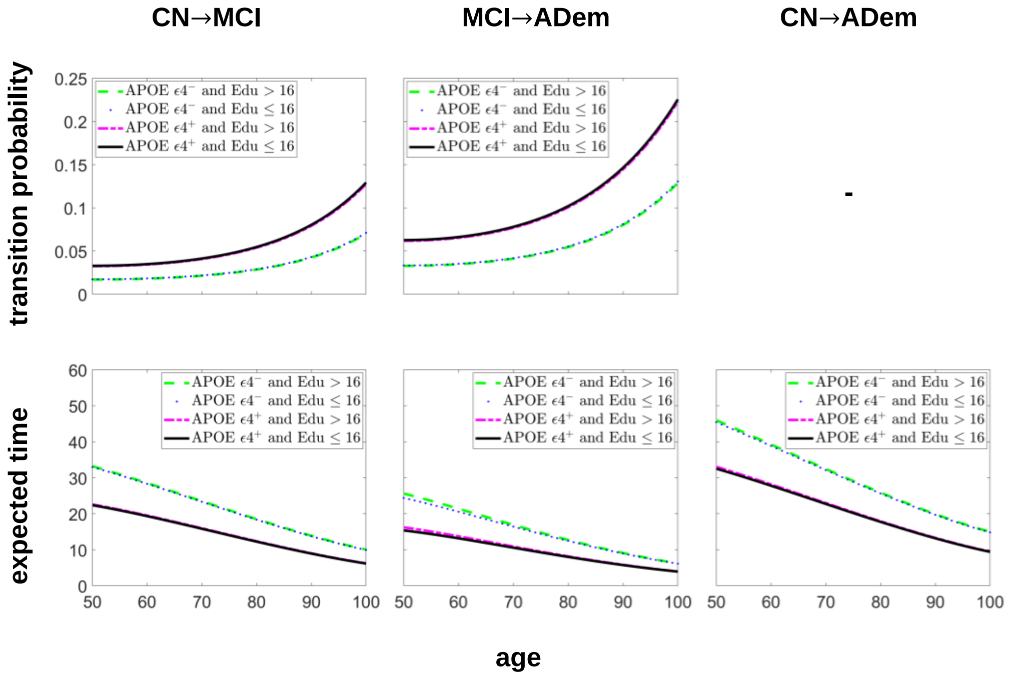

Fig. S8. Probabilities and times to a more severe clinical state: the effect of educational attainment in amyloid-positive individuals. Mean probabilities of transitioning to a more severe state within one year (top), and the expected time to reach a more severe state (bottom), as a function of age in different risk groups defined by the educational level and APOE ε4 status. Edu = Education.

Cognitive markers

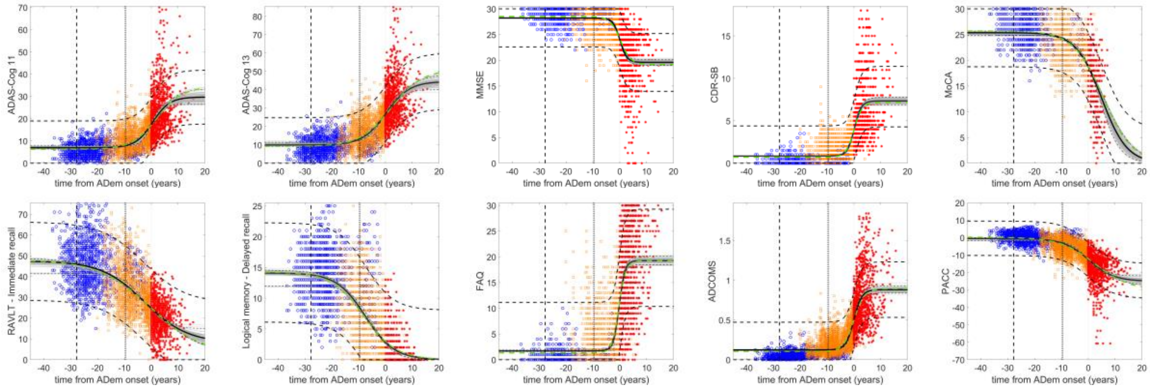

CSF markers

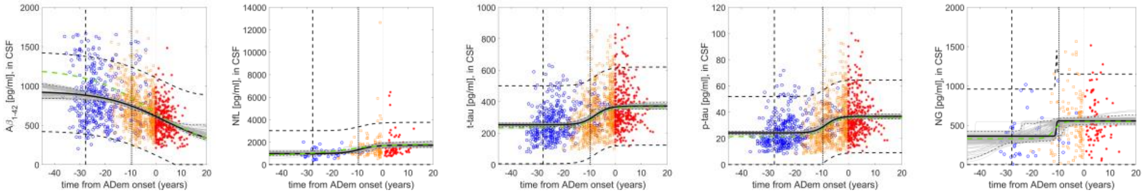

Plasma fluid markers

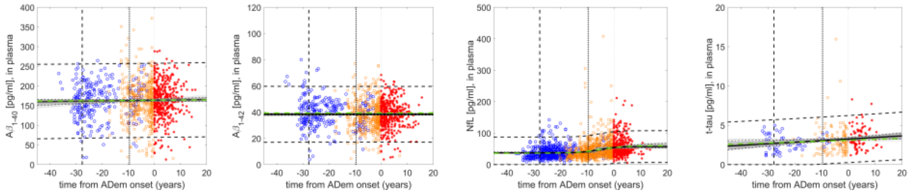

Neuroimaging markers

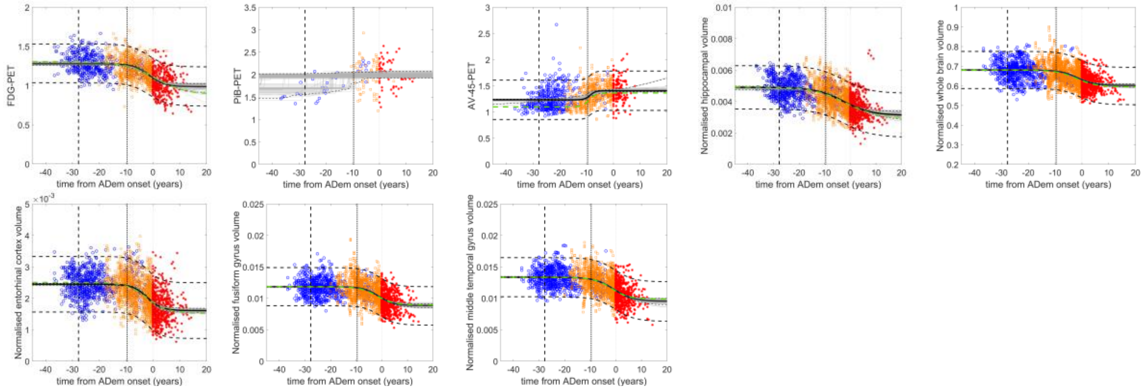

Fig. S9. Biomarker trajectories in amyloid-positive individuals. Trajectories of cognitive, CSF, plasma and neuroimaging markers in individuals with evidence of Aβ accumulation in their brain. The black solid line represents the best fit of the sigmoid function (linear for $A\beta_{1-40}$, $A\beta_{1-42}$ and t-tau in plasma), the dashed thick lines the 95% prediction interval and the dashed thin lines the estimated 95% bootstrap confidence interval (the 2.5th and 97.5th percentiles for each time point). The light grey lines are the best fit of the function to 100 randomly selected bootstrap samples (out of the 500). The symbols show individual measurements (blue (circles): CN, orange (squares): MCI, red (asterisks): ADem). For comparison, the green dashed line is the best fit of the function to the whole dataset, as presented in Fig. 3. The vertical dashed and thick dotted lines represent the average expected time from ADem onset of individuals that are CN (23.88 years) and MCI (7.43 years) at baseline, respectively.

Table S4. Best-fit for each marker in amyloid-positive individuals (Fig. S9): mean parameter values obtained from the model and 95% confidence interval (in brackets) computed using 500 bootstrap samples.

	Parameter estimates			
	r_1	r_2	r_3	r_4
**Cognitive markers**				
ADAS-Cog 11	7.13 (6.45, 7.81)	29.49 (26.45, 33.72)	-0.35 (-0.57, -0.23)	0.52 (-0.20, 1.83)
ADAS-Cog 13	9.96 (9.23, 11.41)	44.52 (40.02, 48.61)	-0.23 (-0.34, -0.19)	0.27 (-0.58, 1.20)
MMSE	28.22 (28.13, 28.30)	19.63 (19.01, 20.18)	-0.79 (-0.94, -0.62)	0.08 (-0.15, 0.35)
CDR-SB	0.84 (0.79, 0.89)	7.34 (6.90, 7.76)	-0.76 (-0.9, -0.61)	0.17 (-0.06, 0.41)
MoCA	25.28 (24.86, 25.67)	0.00 (0.00, 0.00)	-0.22 (-0.27, -0.18)	4.90 (4.20, 5.69)
RAVLT_Immediate	47.17 (41.66, 49.21)	7.27 (1.48, 15.00)	-0.12 (-0.26, -0.10)	-2.00 (-3.47, 0.09)
LM_Delayed	14.09 (12.02, 14.57)	0.00 (0.00, 0.00)	-0.19 (-0.25, -0.17)	-7.45 (-8.29, -5.19)
FAQ	1.71 (1.59, 1.86)	19.25 (18.45, 20.18)	-0.92 (-1.10, -0.74)	-0.20 (-0.35, -0.03)
ADCOMS	0.12 (0.12, 0.13)	0.88 (0.84, 0.93)	-0.68 (-0.81, -0.54)	0.00 (-0.21, 0.26)
PACC	-0.52 (-1.77, -0.03)	-24.96 (-27.68, -21.91)	-0.22 (-0.32, -0.18)	0.20 (-0.58, 1.16)
**CSF markers**				
$A\beta_{1\text{-}42}$	954.72 (841.69, 1106.40)	269.07 (203.00, 500.22)	-0.10 (-0.19, -0.05)	-0.37 (-6.10, 5.54)
NfL	980.04 (750.32, 1191.60)	1879.73 (1538.30, 2347.80)	-1.35 (-13.35, -0.07)	-7.68 (-15.25, 2.08)
t-tau	252.81 (239.71, 264.08)	372.17 (352.88, 396.30)	-0.76 (-2.32, -0.20)	-8.06 (-10.21, -5.89)
p-tau	24.17 (22.98, 25.35)	36.70 (34.99, 38.73)	-1.32 (-4.74, -0.23)	-8.68 (-11.01, -6.74)
Ng	306.75 (6.10, 422.56)	568.25 (515.24, 661.51)	-6.27 (-35.37, -0.08)	-14.03 (-31.52, -5.91)
**Plasma fluid markers**				
$A\beta_{1\text{-}40}$	163.67 (160.88, 166.52)	0.09 (0.00, 0.30)	-	-
$A\beta_{1\text{-}42}$	38.39 (37.69, 39.00)	0.00 (0.00, 0.01)	-	-
NfL	37.70 (35.59, 39.94)	58.84 (52.79, 68.20)	-1.56 (-14.30, -0.18)	-4.67 (-7.96, -0.61)
t-tau	3.25 (3.06, 3.46)	0.02 (0.00, 0.03)	-	-
**Neuroimaging markers**				
FDG-PET	1.28 (1.26, 1.30)	0.99 (0.90, 1.03)	-0.34 (-0.57, -0.18)	-1.40 (-2.43, 0.80)
PiB-PET	1.74 (1.48, 2.00)	1.99 (1.93, 2.07)	-25.04 (-48.88, -2.53)	-33.25 (-92.87, 34.48)
AV-45-PET	1.23 (1.21, 1.25)	1.41 (1.39, 1.42)	-1.74 (-6.07, -0.24)	-8.89 (-10.48, -7.73)
Hippocampus	0.0049 (0.0047, 0.0050)	0.0031 (0.0028, 0.0034)	-0.20 (-0.31, -0.15)	-2.81 (-4.44, -0.59)
Whole brain	0.68 (0.6774, 0.6839)	0.60 (0.59, 0.61)	-0.31 (-0.39, -0.24)	-1.66 (-2.62, -0.53)
Entorhinal cortex	0.0024 (0.0024, 0.0025)	0.0016 (0.0015, 0.0017)	-0.38 (-0.53, -0.30)	-2.21 (-2.89, -1.54)
Fusiform gyrus	0.0118 (0.0117, 0.0119)	0.0088 (0.0085, 0.0092)	-0.34 (-0.42, -0.27)	-1.26 (-2.11, -0.26)
Middle temporal gyrus	0.0134 (0.0132, 0.0135)	0.0095 (0.0089, 0.0100)	-0.27 (-0.34, -0.21)	-0.78 (-1.81, 0.64)
